# Superior interfacial thermal conductance between *β*-Ga_2_O_3_ and diamond realized through metal-assisted epitaxial strategy

**DOI:** 10.1093/nsr/nwag308

**Published:** 2026-05-27

**Authors:** Wentao Huang, Yuehui Li, Tianqi Bai, Jing Zhou, Xing Li, Jie Zhu, Ying Guo, Longbin Yan, Weiwei Yan, Bingqi Linghu, Lingrao Fu, Xiaodong Wang, Jiaojiao Sun, Huanjie Yang, Shaobo Cheng, Peng Gao, Chongxin Shan

**Affiliations:** Henan Key Laboratory of Diamond Materials and Devices, Key Laboratory of Integrated Circuit, Ministry of Education, School of Physics, Zhengzhou University, Zhengzhou 450052, China; Institute of Quantum Materials and Physics, Henan Academy of Sciences, Zhengzhou 450046, China; International Center for Quantum Materials, and Electron Microscopy Laboratory, School of Physics, Peking University, Beijing 100871, China; Academy for Advanced Interdisciplinary Studies, Peking University, Beijing 100871, China; Information Materials Research Department, Suzhou Laboratory, Suzhou 215123, China; Henan Key Laboratory of Diamond Materials and Devices, Key Laboratory of Integrated Circuit, Ministry of Education, School of Physics, Zhengzhou University, Zhengzhou 450052, China; School of Energy and Power Engineering, Key Lab of Ocean Energy Utilization and Energy Conservation of Ministry of Education, Dalian University of Technology, Dalian 116024, China; Henan Key Laboratory of Diamond Materials and Devices, Key Laboratory of Integrated Circuit, Ministry of Education, School of Physics, Zhengzhou University, Zhengzhou 450052, China; Henan Key Laboratory of Diamond Materials and Devices, Key Laboratory of Integrated Circuit, Ministry of Education, School of Physics, Zhengzhou University, Zhengzhou 450052, China; Henan Key Laboratory of Diamond Materials and Devices, Key Laboratory of Integrated Circuit, Ministry of Education, School of Physics, Zhengzhou University, Zhengzhou 450052, China; School of Energy and Power Engineering, Key Lab of Ocean Energy Utilization and Energy Conservation of Ministry of Education, Dalian University of Technology, Dalian 116024, China; School of Energy and Power Engineering, Key Lab of Ocean Energy Utilization and Energy Conservation of Ministry of Education, Dalian University of Technology, Dalian 116024, China; Henan Key Laboratory of Diamond Materials and Devices, Key Laboratory of Integrated Circuit, Ministry of Education, School of Physics, Zhengzhou University, Zhengzhou 450052, China; Henan Key Laboratory of Diamond Materials and Devices, Key Laboratory of Integrated Circuit, Ministry of Education, School of Physics, Zhengzhou University, Zhengzhou 450052, China; Henan Key Laboratory of Diamond Materials and Devices, Key Laboratory of Integrated Circuit, Ministry of Education, School of Physics, Zhengzhou University, Zhengzhou 450052, China; Henan Key Laboratory of Diamond Materials and Devices, Key Laboratory of Integrated Circuit, Ministry of Education, School of Physics, Zhengzhou University, Zhengzhou 450052, China; Institute of Quantum Materials and Physics, Henan Academy of Sciences, Zhengzhou 450046, China; International Center for Quantum Materials, and Electron Microscopy Laboratory, School of Physics, Peking University, Beijing 100871, China; Tsientang Institute for Advanced Study, Hangzhou 310024, China; Henan Key Laboratory of Diamond Materials and Devices, Key Laboratory of Integrated Circuit, Ministry of Education, School of Physics, Zhengzhou University, Zhengzhou 450052, China

**Keywords:** transmission electron microscopy, *β*-Ga_2_O_3_/diamond interface, interfacial phonon mode, Ga etching-assisted epitaxy

## Abstract

*β*-Ga_2_O_3_ exhibits great potential for next-generation power electronics, while its low thermal conductivity poses a challenge to efficient heat dissipation. We address this challenge by developing a gallium-assisted epitaxial strategy to synthesize highly oriented *β*-Ga_2_O_3_ film on diamond. The *β*-Ga_2_O_3_ presents a high thermal conductivity of 9.0 W m^−1^ K^−1^ and low thermal boundary resistance of 6.05 m^2^ K GW^−1^. *In-situ* experiments reveal a high interfacial bonding strength (>2.09 GPa), resulting from the atomically sharp and covalently bonded interface. The identified new interfacial phonon mode at ∼60 meV through the vibrational electron energy-loss spectroscopy can significantly enhance the phonon transport between diamond and *β*-Ga_2_O_3_. The developed gallium-assisted strategy may mitigate the thermal constraints of *β*-Ga_2_O_3_, offering a promising route for the heterointegration of *β*-Ga_2_O_3_-based power devices with diamond.

## Teaser

Gallium-assisted epitaxy enables highly oriented *β*-Ga₂O₃ growth on diamond with low thermal boundary resistance and strong interfacial bonding strength.

## INTRODUCTION

**Figure 1.**
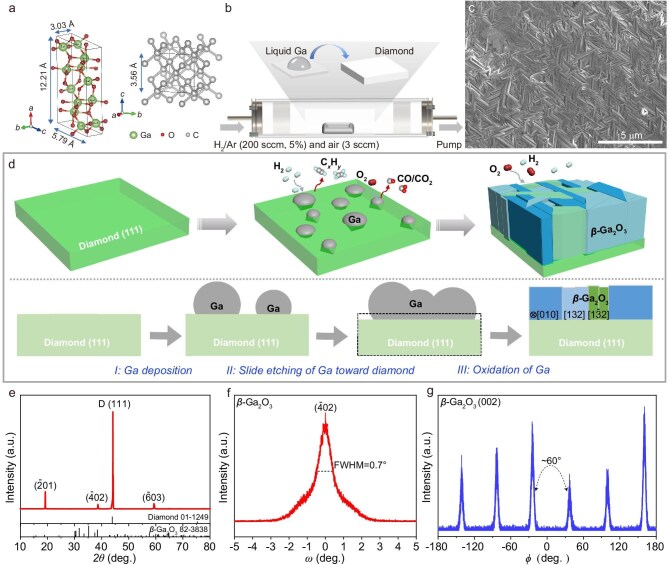
Ga-assisted epitaxial process and structural characterization of *β*-Ga_2_O_3_. Schematic illustrations of (a) the crystal structure of *β*-Ga_2_O_3_ and diamond, and (b) the Ga-assisted chemical vapor deposition (CVD) epitaxial process. (c) Morphology of the synthesized film. (d) Schematic illustration showing the epitaxial process and mechanism. (e) X-ray diffraction (XRD) 2*θ* scan of the *β*-Ga_2_O_3_ film on a diamond (111) substrate. (f) XRD rocking curve of the *β*-Ga_2_O_3_  $(\bar{4}02)$ reflection. (g) XRD *Φ*-scan for the (002) plane of the *β*-Ga_2_O_3_ film.

As an ultra-wide bandgap semiconductor, beta-phase gallium oxide (*β*-Ga_2_O_3_) has shown great potential for advanced power devices due to its high breakdown field [1]. Since its relatively low thermal conductivity (*κ*) (∼10–27 W m^−1^ K^−1^) can limit the maximum power density and accelerate device degradation, thermal management has been one of the bottleneck issues in *β*-Ga_2_O_3_-based power devices [2]. With an exceptionally high *κ* (∼2200 W m^−1^ K^−1^), diamond has been extensively regarded as an ideal material for heat dissipation [3]. As a near-junction cooling strategy, directly integrating *β*-Ga_2_O_3_ onto diamond substrate can efficiently spread heat away from the hot spots in the power devices. Nevertheless, the large lattice mismatch between *β*-Ga_2_O_3_ (monoclinic, *a* = 12.21 Å, *b* = 3.03 Å, *c* = 5.79 Å, *β* = 103.8°) and diamond (cubic, *a* = 3.56 Å) (Fig. 1a) has made the construction of high quality heterointerface quite challenging. Current growth approaches, typically based on atomic layer deposition (ALD) and pulsed laser deposition (PLD), generally lead to unsatisfied crystal quality and the appearance of other Ga_2_O_3_ phases (*ε*- or *γ*-) at the interface [4–6]. Current *β*-Ga_2_O_3_/diamond bonding approaches, often based on van der Waals bonding and hydrophilic bonding, remain constrained by the low bonding strength [7,8]. Therefore, developing novel strategies toward the construction of high quality *β*-Ga_2_O_3_/diamond heterointerface is of great importance.

Additionally, as the size of electronic device approaches the mean free path of hot carriers, thermal boundary conductance (TBC) becomes a significant factor in heat transfer, acting as a bottleneck that limits performance improvement [9]. On one hand, the TBC and interface phonon transmission can be greatly affected by the interface morphology, interlayer, and the interface bonding strength [4,10]. When diamond is bonded with other semiconductors, the considerable disparity in their phonon density of states can result in decreased TBC [11]. On the other hand, the interfacial bonding strength significantly impacts the heat transfer efficiency. Strong chemical bonds, such as covalent bonds, can promote better heat conduction, while weak van der Waals forces or other non-covalent interactions can restrict heat flow [12]. Additionally, the large thermal expansion coefficient mismatch can induce substantial thermal stress (up to GPa) at the bonded interface by the hot spots [2,13] and lead to interface failure issues of power devices [14]. Hence, for efficient heat dissipation and reliability of *β*-Ga_2_O_3_ based power devices, constructing *β*-Ga_2_O_3_/diamond heterostructures with atomicly flat and covalently bonded interfacial structure is of fundamental importance.

Herein, we have developed a Ga-assisted epitaxy technique by using chemical vapor deposition (CVD) process. Adopting the interfacial reaction between Ga and diamond at high temperature, we realized atomic-scale epitaxial growth of $(\bar{2}01)$-orientated *β*-Ga_2_O_3_ films on (111) diamond. Interfacial characterizations confirmed that the *β*-Ga_2_O_3_$(\bar{2}01)$ plane is covalently bonded to the diamond (111) plane through the C−O bonds, resulting in an ultra-high interfacial fracture strength (>2.09 GPa). Using the vibrational electron energy-loss spectroscopy (EELS) technique, we identified a novel interfacial phonon mode at ∼60 meV, which efficiently mediates heat transfer and can significantly enhance the TBC. The high TBC value (165.4 MW m^−2^ K^−1^) was subsequently confirmed by time-domain thermoreflectance (TDTR) measurements. Our work presents a novel strategy for constructing high-quality *β*-Ga_2_O_3_/diamond heterostructures with efficient thermal transport, and provides critical insights into the phonon-driven thermal transport mechanisms across the *β*-Ga_2_O_3_/diamond interface.

## RESULTS AND DISCUSSIONS

### Ga-assisted heteroepitaxy of *β*-Ga_2_O_3_ films on diamond

Metals can facilitate the diamond-to-graphite transition at high temperatures, thereby creating coherent interfaces with diamond {111} planes [15–17]. By utilizing the ‘slide etching’ behavior of metal on diamond (111) surface, continuous film with atomic flat interface can be constructed on diamond (111) substrate. Here, liquid Ga was used as the precursor to grow *β*-Ga_2_O_3_ on diamond (111) substrate with CVD process (Fig. 1b). The obtained films cover the entire diamond surface (3 mm × 3 mm) (Fig. 1c and Fig. S1). Figure 1d presents the proposed epitaxial mechanisms. First, metallic Ga was deposited onto the diamond surface (Ⅰ), initiating a transformation of the diamond into graphite at 1000°C through the interfacial reaction. The resulting graphite can then be converted to C*_x_*H*_y_*/CO/CO_2_ and removed by carrier gas (H_2_/Ar and air). The slide etching behavior of metals on the diamond (111) surface promotes the formation of an atomic flat interfacial structure (Ⅱ) [15,17]. Finally, the deposited Ga can be oxidized to form Ga_2_O_3_ on freshly etched diamond surface by the O_2_, and transformed into *β*-Ga_2_O_3_ under the 1000°C (Fig. 1d) (Ⅲ) [18].

It should be mentioned that, at high temperatures, diamond can also suffer oxidative etching process in O_2_ atmosphere [19,20]. Along with the diamond-to-graphite transition, the graphite was then reacted with O_2_ and transformed to CO and CO_2_ [21,22], leaving a clean surface for the oxidation of Ga into *β*-Ga_2_O_3_ (Fig. 1d). Therefore, the oxidative etching of diamond didn’t affect the initial nucleation of *β*-Ga_2_O_3_. For the growth of *β*-Ga_2_O_3_, the low-O_2_ growth and the H_2_ annealing can assist the removal of oxygen vacancies in *β*-Ga_2_O_3_, resulting in high-quality *β*-Ga_2_O_3_ film [23].

X-ray diffraction (XRD) scan (Fig. 1e) reveals prominent diffraction peaks corresponding to $(\bar{2}01)$, $(\bar{4}02)$, and $(\bar{6}03)$ planes of the monoclinic *β*-Ga_2_O_3_ and (111) plane of diamond, indicating the out-of-plane epitaxial relationship of *β*-Ga_2_O_3_$(\bar{2}01)$/diamond (111). The enlarged view of the $(\bar{2}01)$ peak (Fig. S2a) presents a full width at half maximum (FWHM) value of ∼0.08^o^. The obtained rocking curve concerning the $({\mathrm{\bar{4}}}0{\mathrm{2}})$ peak of *β*-Ga_2_O_3_ (Fig. 1f) shows a FWHM value of ∼0.7^o^, which is much narrower than other typically reported heteroepitaxial *β*-Ga_2_O_3_ films on diamond [14,24–27] (Fig. S2b). In our synthesis process, it can be seen that the introduced H_2_ (200 sccm, 5% H_2_/Ar) and O_2_ have greatly improved the quality of *β*-Ga_2_O_3_ film. In the acquired phi (*Φ*) scan of the (002) plane of *β*-Ga_2_O_3_ (Fig. 1g), six diffraction peaks are observed ∼60^o^ apart, demonstrating that the approximate sixfold symmetry present in the epitaxial *β*-Ga_2_O_3_ film [26,28]. The acquired Raman spectrum (Fig. S2c) shows 11 vibrational peaks at 111, 142, 167, 197, 317, 343, 416, 473, 629, 653, and 765 cm^−1^, which correspond well with the Raman peaks of *β*-Ga_2_O_3_.

**Figure 2.**
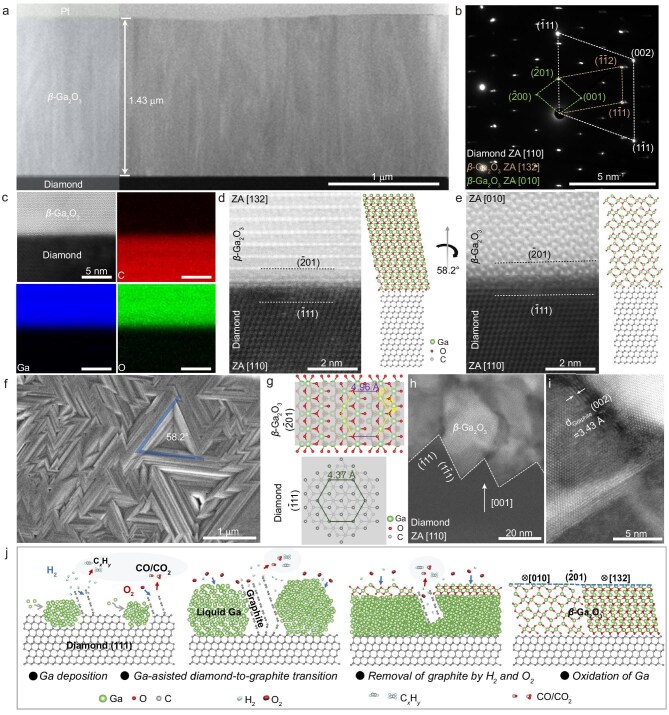
Interfacial structure relationship and the heteroepitaxial mechanism of *β*-Ga_2_O_3_ on diamond. (a) The cross-sectional high angle annular dark field scanning transmission electron microscopy (HAADF-STEM) image. (b) The selected area electron diffraction (SAED) and (c) the energy-dispersive X-ray spectroscopy (EDX) mapping of the *β*-Ga_2_O_3_/diamond heterostructure. Atomically resolved HAADF-STEM images of the interface with orientation relationship of (d) *β*-Ga_2_O_3_$(\bar{2}01)$[132]//diamond$(\bar{1}11)$[110] and (e) *β*-Ga_2_O_3_$(\bar{2}01)$[010]//diamond$(\bar{1}11)$[110]. Corresponding atom arrangements of *β*-Ga_2_O_3_ and diamond are also presented. (f) Surface morphology of the as-grown *β*-Ga_2_O_3_ on diamond (111). (g) Schematic illustration showing the atom arrangement in the $(\bar{2}01)$ plane of *β*-Ga_2_O_3_ and the $(\bar{1}11)$ plane of diamond. (h and i) Interfacial structure characterizations of *β*-Ga_2_O_3_ growth on diamond (001) substrate. (j) Atomic-scale illustrations of the growth of *β*-Ga_2_O_3_ through metal-assisted epitaxial strategy.

The cross-sectional high angle annular dark field scanning transmission electron microscopy (HAADF-STEM) characterization reveals that the *β*-Ga_2_O_3_ film is ∼1.43 μm in thickness and consists of columnar grains (Fig. 2a). It should be mentioned that both the inner and edge regions of the polished diamond (111) substrate possess atomic flat interface with directly bonded *β*-Ga_2_O_3_ (Fig. S3). The corresponding selected area electron diffraction (SAED) patterns (Fig. 2b and Fig. S4) exhibit two differently oriented *β*-Ga_2_O_3_ grains in the epitaxial film, with the epitaxial relationships determined as *β*-Ga_2_O_3_$(\bar{2}01)$[132]//diamond$(\bar{1}11)$[110] and *β*-Ga_2_O_3_$(\bar{2}01)$[010]//diamond$(\bar{1}11)$[110], respectively. It is worth noting that in both grains, the *β*-Ga_2_O_3_$(\bar{2}01)$ is parallel to the diamond$(\bar{1}11)$, corresponding well with our XRD characterization (Fig. 1e). According to the interfacial structural characterizations (Fig. S5a–e), *β*-Ga_2_O_3_$(\bar{2}01)$ forms an atomically flat interface with diamond $(\bar{1}11)$ without interfacial layer. The energy-dispersive X-ray spectroscopy (EDX) mapping and EELS further confirm the uniform distribution of C, Ga, and O in diamond and *β*-Ga_2_O_3_ (Fig. 2c and Fig. S6).

### Ga-assisted epitaxial mechanism

To confirm the proposed growth mechanism (Fig. 1d), the interfacial structures between diamond and *β*-Ga_2_O_3_ were scrutinized. Figure 2d and e presents the typical atomic structures of the two types of *β*-Ga_2_O_3_ grains on diamond (111), that is, *β*-Ga_2_O_3_$(\bar{2}01)$[132]//diamond$(\bar{1}11)$[110] (Fig. 2d) and *β*-Ga_2_O_3_$(\bar{2}01)$[010]//diamond$(\bar{1}11)$[110] (Fig. 2e). Notably, at the interface, the oxygen (O) atoms in *β*-Ga_2_O_3_ directly form covalent bonds with the carbon (C) atoms in diamond (Fig. 2d and e and Fig. S5f–i). When rotating around the direction perpendicular to $(\bar{2}01)$ plane, the rotation angle of *β*-Ga_2_O_3_ [132] with [010] and $[1\bar{3}2]$ is 58.2^o^ and 60.9^o^ (Fig. 2d and e and Fig. S7). Therefore, the *β*-Ga_2_O_3_ grains form in-plane rotational domains with angles of ∼60^o^ in the as-grown film (Figs 1c and d and 2f). Additionally, the C atoms arrangement on the diamond (111) is a symmetric hexagonal shape with a side distance of 4.37 Å, and the O atoms arrangement on the *β*-Ga_2_O_3_$(\bar{2}01)$ is an asymmetric hexagonal shape with two different side distances of 4.96 and 5.15 Å (Fig. 2g and Fig. S8) [6,25,26]. The similarity in atom arrangement, along with the relatively small lattice mismatch (13.5%–17.76%), creates favorable conditions for the highly oriented heteroepitaxial growth of *β*-Ga_2_O_3_ on diamond (111).

To confirm the etching behavior of Ga toward diamond during the CVD process, we also utilized polished diamond (001) substrates to grow *β*-Ga_2_O_3_. As presented in Fig. 2h, the initially flat diamond (001) surface has evolved into a zigzag morphology, exposing diamond {111} planes, which are directly bonded to the *β*-Ga_2_O_3_ grains. Additionally, graphite formation was observed at the *β*-Ga_2_O_3_ grain boundary (Fig. 2i). These phenomena further verified the Ga-assisted etching of diamond during the growth process (Fig. 1d). Figure 2j summarizes the atomic-scale evolution process during the epitaxial growth of *β*-Ga_2_O_3_, including the Ga deposition, Ga-assisted diamond-to-graphite transition, removal of graphite by H_2_ and O_2_, and the oxidation of Ga. It should be mentioned that high temperature (1000°C) is essential to drive the etching behavior of Ga toward diamond, while low temperature (600°C) can lead to the growth-stress-induced nano-crystallization of diamond surface (Fig. S9). The surface roughness is another important factor for the continuous growth of *β*-Ga_2_O_3_ films (Fig. S10).

### Ultra-high interfacial bonding strength

**Figure 3.**
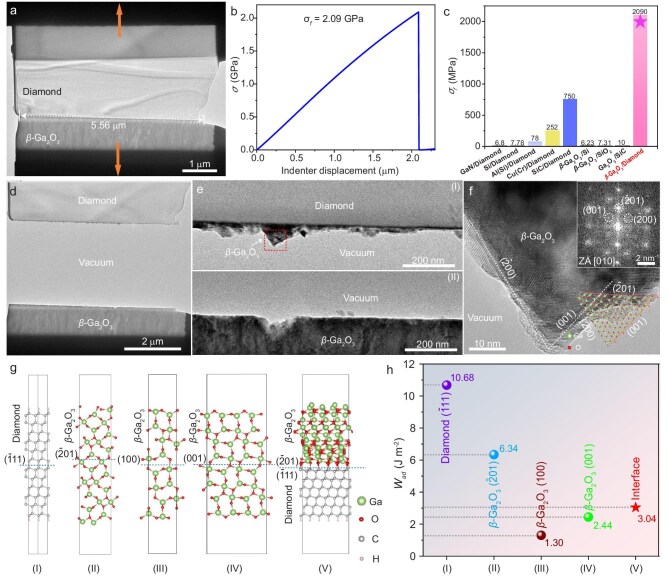
Bonding strength measurement of the *β*-Ga_2_O_3_/diamond interface. (a) Transmission electron microscopy (TEM) image of the focused ion beam (FIB)-fabricated *β*-Ga_2_O_3_/diamond sample for the tensile test. (b) Relationship between the applied stress and the indenter displacement. (c) The comparison of the fracture strength (*σ_f_*) of distinct diamond (and Ga_2_O_3_)-based heterostructures. (d) The low-mag and (e) high-mag TEM images presenting the fracture morphology. (f) High-resolution TEM (HRTEM) image of the remained *β*-Ga_2_O_3_ indicated by the frame in (e(I)). Inset shows the corresponding fast Fourier transform (FFT) pattern. (g) Side view models and (h) the corresponding calculated work of adhesion (*W_ad_*) for (I) diamond $(\bar{1}11)$, (II) *β*-Ga_2_O_3_$(\bar{2}01)$, (III) *β*-Ga_2_O_3_ (100), (IV) *β*-Ga_2_O_3_ (001), and (V) *β*-Ga_2_O_3_$(\bar{2}01)$**/**diamond$(\bar{1}11)$ interface. The dashed line marks the fracture position.

To assess the bonding strength of the *β*-Ga_2_O_3_/diamond interface, we conducted an *in-situ* transmission electron microscopy (TEM) tensile test using a push-to-pull (PTP) device (Figs S11a and S12 and Supplementary Movie). The *β*-Ga_2_O_3_/diamond sample with a length of 5.56 μm and a thickness of 167.9 nm was fabricated, and the interface can be clearly observed (Fig. 3a and Fig. S11b). Figure 3b presents the relationship between the tensile stress and indenter displacement. The heterostructure underwent a brittle fracture (Fig. 3b) at 2.09 GPa, exceeding the fracture strength (*σ*_f_) of most previously reported diamond (and Ga_2_O_3_)-based heterostructures (Fig. 3c and Table S1) [29–36]. The monocrystalline *β*-Ga_2_O_3_ has a primary (100) cleavage plane and a secondary (001) cleavage plane [37,38]. The corresponding fractured morphology (Fig. 3d and e and Fig. S13) showed that the fracture mostly occurred on the *β*-Ga_2_O_3_ side, exhibiting a zigzag morphology with sharp $(\bar{2}00)$ and (001) cleavage planes (Fig. 3f). This indicates that the interfacial C−O covalent bonds possess a higher strength than certain types of Ga–O bonds in *β*-Ga_2_O_3_. It should be mentioned that this zigzag fracture morphology also occurred in the $(\bar{2}01)$ oriented *β*-Ga_2_O_3_ polycrystalline film deposited on 4H-SiC substrate (Fig. S14), indicating that this morphology should be a universal result of $(\bar{2}01)$ oriented *β*-Ga_2_O_3_ polycrystalline film.

First principal calculations were further conducted to quantificationally compare the energy needed to separate the two bonded planes (work of adhesion, *W_ad_*) [39,40]. As presented in Fig. 3g and h, the *W_ad_* of the constructed diamond $(\bar{1}11)$ planes (I), *β*-Ga_2_O_3_$(\bar{2}01)$ planes (II), *β*-Ga_2_O_3_ (100) planes (III), *β*-Ga_2_O_3_ (001) planes (IV), and *β*-Ga_2_O_3_$(\bar{2}01)$/diamond $(\bar{1}11)$ heterointerface (V) are 10.68, 6.34, 1.30, 2.44, and 3.04 J/m^2^, respectively. A larger *W_ad_* makes it more difficult to separate the interface. According to our calculations, the bonding strength is as follows: C–C (diamond $(\bar{1}11)$) > Ga–O (*β*-Ga_2_O_3_$(\bar{2}01)$) > C–O (*β*-Ga_2_O_3_/diamond) > Ga–O (*β*-Ga_2_O_3_ (001)) > Ga–O (*β*-Ga_2_O_3_ (100)). The higher *W_ad_* of the heterostructure compared to that of *β*-Ga_2_O_3_ (100) and (001) leads to preferred fracture at the *β*-Ga_2_O_3_ side along the (100) and (001) planes.

### Efficient thermal transport across the *β*-Ga_2_O_3_/diamond interface

**Figure 4.**
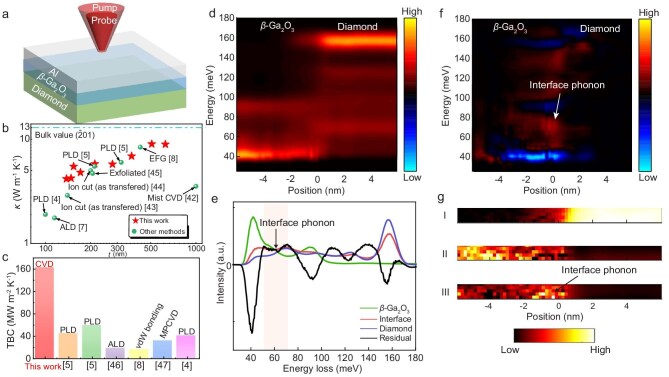
Time-domain thermoreflectance (TDTR) and phonon measurement at the *β*-Ga_2_O_3_$(\bar{2}01)$/diamond$(\bar{1}11)$ heterostructure. (a) Schematic of the TDTR based on a pump and probe technique measuring the thermal boundary conductance (TBC) of the interface. Comparison of (b) *κ* of *β*-Ga_2_O_3_ and (c) TBC of the Ga_2_O_3_/diamond interface between this work and previously reported values. (d) The measured electron energy-loss spectral mapping across the interface. The interface is labeled as zero position. (e) Phonon spectra and interface vibrational modes. (f) The measured electron energy-loss spectroscopy (EELS) line profile of the fitting residual. (g) The non-negative matrix factorization (NMF) intensity map for components I, II, and III, respectively.

The high structural quality and bonding strength of the constructed *β*-Ga_2_O_3_/diamond interface can lead to a good heat dissipation capability. To confirm this, we acquired the *κ* of the *β*-Ga_2_O_3_ and the TBC of the* β*-Ga_2_O_3_/diamond interface through the TDTR measurements (Fig. 4a and Fig. S15). Bulk *β*-Ga_2_O_3_ single crystal possesses anisotropic *κ* values [41]. Compared with the reported *κ* values of Ga_2_O_3_ [4,5,7,8,42–45], the overall *κ* of our $(\bar{2}01)$-oriented *β*-Ga_2_O_3_ film is relatively high and reaches 9.0 W m^−1^ K^−1^ at a thickness of 509 nm (Fig. 4b). Despite the large *κ* difference between diamond and *β*-Ga_2_O_3_, the constructed *β*-Ga_2_O_3_/diamond interface presents a higher TBC value of 165.4 MW m^−2^ K^−1^ (Fig. 4c) than the previously reported Ga_2_O_3_/diamond interface [4,5,8,46,47].

### Atomic-scale insights into phonon-driven thermal transport

The microscopic thermal transport mechanism is further investigated by the vibrational EELS technique, which is used to reveal the interface phonons and their impact on the TBC of the *β*-Ga_2_O_3_/diamond heterointerface [9]. The EEL spectral mapping and line profile across the *β*-Ga_2_O_3_$(\bar{2}01)$/diamond$(\bar{1}11)$ interface is provided in Fig. 4d and Fig. S16, respectively. The calculated phonon dispersion curves of bulk diamond and *β*-Ga_2_O_3_ are presented in Fig. S17. The phonon spectra exhibit intensity on both sides below ∼100 meV, corresponding to extended modes [11], which act as phonon elastic scattering channels to contribute to interfacial heat flow. However, a significant phonon mismatch is observed above ∼100 meV, with diamond providing acoustic branches (∼125 meV) and optical branches (∼158 meV), while *β*-Ga_2_O_3_ lacking phonon modes in this window, corresponding to partially extended modes [48].

To isolate the interfacial vibrational signature, the phonon spectra evolution is further analyzed through the spectra of bulk *β*-Ga_2_O_3_ (green), bulk diamond (blue), interface (red), and the residual spectrum (black) (Fig. 4e). The interface EEL spectrum exhibits a new peak at ∼60 meV (highlighted by arrow in Fig. 4e), indicating the emergence of a new interfacial phonon. This peak is further supported by a pronounced positive residual between 50 and 80 meV (Fig. 4f). Figure 4f shows that the interface mode propagates farther on the *β*-Ga_2_O_3_ side than on the diamond side, which can be attributed to the Coulomb interaction of ionic Ga-O bonding, in contrast to the nonpolar C–C bonding. This interpretation is further supported by the following calculated eigenvector. As a powerful tool to extract interpretable characteristics from the EEL spectra data [48,49], the non-negative matrix factorization (NMF) is employed to analyze the entire spectrum image, enabling the separation of intrinsic spectra from the interface [49]. As presented in Fig. 4g, three distinct components are found. Components I and II are spatially confined at the diamond and *β*-Ga_2_O_3_ layers, respectively, and thus are attributed to vibrational signals from diamond and *β*-Ga_2_O_3_. Component III is highly confined to a 1 nm slab centered on the interface, confirming its interfacial origin, which can facilitate efficient phonon transfer at the interface. Figure S18 presents the extracted EEL spectra of components I, II, and III by NMF decomposition, respectively.

**Figure 5.**
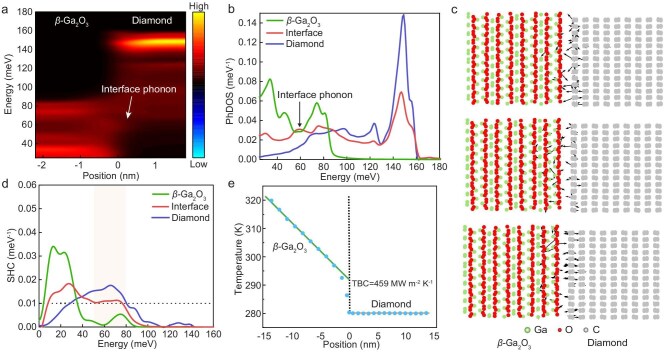
The calculated interface phonon of the *β*-Ga_2_O_3_$(\bar{2}01)$/diamond$(\bar{1}11)$ heterostructure. (a) The calculated phonon density of states (PhDOS) across the *β*-Ga_2_O_3_$(\bar{2}01)$/diamond$(\bar{1}11)$ interface. (b) The extracted PhDOS of bulk *β*-Ga_2_O_3_, bulk diamond, and interface. (c) Phonon eigenvectors for three typical localized interface modes with energies of 61.5, 61.8, and 59.5 meV, respectively. (d) The spectral distribution of heat current (normalized to unity) quantifies the percentage contribution of phonons across different energy ranges to the total heat transport at bulk *β*-Ga_2_O_3_, bulk diamond, and interface. (e) Temperature profiles across the interface under nonequilibrium molecular dynamics (NEMD) simulations.

To reveal the influence of the interfacial phonon mode on the thermal transfer behavior, we performed phonon calculations using Graphics Processing Units Molecular Dynamics (GPUMD) [50] on the constructed *β*-Ga_2_O_3_$(\bar{2}01)$**/**diamond$(\bar{1}11)$ model (see Materials and Methods). Figure 5a presents the calculated phonon density of states (PhDOS) across the *β*-Ga_2_O_3_$(\bar{2}01)$**/**diamond$(\bar{1}11)$ interface. A distinct spectral peak near 60 meV (highlighted by the white arrow) is localized near the interface. The calculated PhDOS of bulk *β*-Ga_2_O_3_ (green), * β*-Ga_2_O_3_/diamond interface (red), and bulk diamond (blue) are shown in Fig. 5b. As indicated by the arrow, the spectral feature at ∼60 meV is significantly enhanced at the *β*-Ga_2_O_3_$(\bar{2}01)$**/**diamond$(\bar{1}11)$ interface. The eigenvectors of three typical localized modes (LMs) from side view are illustrated in Fig. 5c, which correspond to coupled out-of-plane motions of C–O bonded atoms and involve vibrations much stronger at the *β*-Ga_2_O_3_/diamond interface, indicating their highly localized nature. Due to the long-range nature of the Coulomb interaction of ionic Ga–O bonding, compared to the short-range nature of nonpolar C–C bonding, atomic vibrations extend across several atomic layers on the* β*-Ga_2_O_3_ side, whereas only one atomic layer on diamond side.

To quantify the contribution of the interfacial phonon mode (∼60 meV) to TBC, we calculated the spectral distribution of heat current (SHC, normalized to unity) using the nonequilibrium molecular dynamics (NEMD) simulations. This analysis quantifies the percentage contribution of phonons across different energy ranges to the total heat transport at different locations. As presented in Fig. 5d, the heat flux in the bulk diamond layer is predominantly facilitated by phonons in the 20–100 meV range. Upon reaching the *β*-Ga_2_O_3_/diamond interface, thermal energy is transferred to phonons in the 0–80 meV range, with a pronounced shoulder centered at 60 meV. As the heat flux propagates through the bulk *β*-Ga_2_O_3_ layer, this thermal energy is further transferred to phonons in the 0–40 meV range. Due to the great contribution to heat current from phonons with energy between 50 and 80 meV (Fig. 5d), the *β*-Ga_2_O_3_$(\bar{2}01)$**/**diamond$(\bar{1}11)$ interface demonstrates a high TBC of 459 MW m^−2^ K^−1^ (Fig. 5e). This compelling evidence confirms that the interfacial modes at the *β*-Ga_2_O_3_$(\bar{2}01)$**/**diamond$(\bar{1}11)$ interface facilitate enhanced inelastic scattering of phonons across the interface, thereby boosting *κ*.

## CONCLUSIONS

In summary, we achieved the atomically epitaxial growth of $(\bar{2}01)$-oriented *β*-Ga_2_O_3_ films on diamond (111) substrate using a CVD method by adopting the interfacial reaction between Ga and diamond. The obtained *β*-Ga_2_O_3_ film presents a high *κ* of 9.0 W m^−1^ K^−1^ and a low thermal boundary resistance (TBR) of 6.05 m^2^ K GW^−1^. The covalent C–O bond at the interface leads to a high interfacial fracture strength over 2.09 GPa. The observed interfacial phonon mode at ∼60 meV provides a phonon transmission channel and significantly improves the TBC. These findings provide insights into the microscopic mechanisms governing phonon transport at the *β*-Ga_2_O_3_/diamond interface, and offer a strategy for designing heterogeneous interfaces with both high interfacial bond strength and superior *κ*, thereby paving the way for facilitating advancements in *β*-Ga_2_O_3_ based power devices by mitigating the low *κ* bottleneck issue of *β*-Ga_2_O_3_.

## METHODS

Detailed methods can be found in supplementary information.

## Supplementary Material

nwag308_Supplemental_File
