# Supplementary material for: Superior interfacial thermal conductance between β-Ga2O3 and diamond realized through metal-assisted epitaxial strategy
Source: Natl Sci Rev. 2026 May 27;13(13):nwag308. doi: 10.1093/nsr/nwag308 (PMC13339106; doi:10.1093/nsr/nwag308)
Supplement: nwag308_Supplemental_File [file nwag308_supplemental_file.pdf]

Supplementary information for

Superior interfacial thermal conductance between β-Ga_2O_3 and diamond realized through metal-assisted epitaxial strategy

Wentao Huang[1,†], Yuehui Li[2,†], Tianqi Bai[3,4,†], Jing Zhou[5,†], Xing Li[1,*], Jie Zhu[6], Ying Guo[1], Longbin Yan[1], Weiwei Yan[1], Bingqi Linghu[6], Lingrao Fu[6], Xiaodong Wang[1], Jiaojiao Sun[1], Huanjie Yang[1], Shaobo Cheng[1,2,*], Peng Gao[3,7,*] and Chongxin Shan[1,*]

[1]Henan Key Laboratory of Diamond Materials and Devices, Key Laboratory of Integrated Circuit, Ministry of Education, School of Physics, Zhengzhou University, Zhengzhou 450052, China;
[2]Institute of Quantum Materials and Physics, Henan Academy of Sciences, Zhengzhou 450046, China;
[3]International Center for Quantum Materials, and Electron Microscopy Laboratory, School of Physics, Peking University, Beijing 100871, China;
[4]Academy for Advanced Interdisciplinary Studies, Peking University, Beijing 100871, China;
[5]Information Materials Research Department, Suzhou Laboratory, Suzhou 215123, China;
[6]School of Energy and Power Engineering, Key Lab of Ocean Energy Utilization and Energy Conservation of Ministry of Education, Dalian University of Technology, Dalian 116024, China;
[7]Tsientang Institute for Advanced Study, Hangzhou 310024, China.

***Corresponding authors.** E-mails: xingli@zzu.edu.cn; chengshaobo@zzu.edu.cn; pgao@pku.edu.cn; cxshan@zzu.edu.cn

[†]Equally contributed to this work.

This file includes:

Materials and Methods

Supplementary Figures 1 to 18

Supplementary Table 1

Supplementary References 1-40

Materials and Methods

The epitaxial of β-Ga_2O_3 on diamond

The β-Ga_2O_3 films were grown on the commercially available diamond substrates (3 mm×3 mm×0.5 mm). First, the diamond surface was polished to surface roughness smaller than 3 nm. Then, the surface graphitization as well as surface contaminants induced during the polishing process were removed by immersing into the aqua regia (HCl: HNO_3=3:1, volume ratio) for more than 3 h. Prior to the growth, the diamond substrates were ultrasonically cleaned in acetone, ethanol, and deionized water for 10 min successively, and blow-dried using high-purity nitrogen.

The diamond substrate was then loaded into a 2 in. quartz tube in the middle of the heating zone in the furnace (OTF-1200), and the high purity Ga metal (⩾ 99.999%, Aladdin) was placed 5 mm apart. The quartz tube was firstly evacuated and then washed with Ar (200 sccm) to expel the remaining air. Then, the 200 sccm H_2/Ar (H_2 5%, 5 N) and 3 sccm air were introduced into the chamber. During the growth process, the furnace was heated to 1000°C (ramp rate: 10°C/min) and maintained for 180 min to deposit β-Ga_2O_3 on diamond. Following the growth process, the quartz tube was cooled to room temperature under continuous H_2/Ar and air flow. The entire device is placed in a fume hood and kept ventilated throughout the experiment.

Material characterizations

The X-ray diffraction (XRD) patterns of the samples were obtained using a diffractometer (X' Pert Pro, PANalytical) in the Bragg-Brentano geometry with the Cu Kα line (λ = 1.5418 Å) as the radiation source, and rocking curve, phi (Φ) scan were examined using an X-ray diffractometer (SmartLab, Rigaku Corp.). The Raman spectra were collected on a confocal Raman spectrometer (Horiba LabRAM HR Evolution) by employing an excitation laser of 532 nm. Scanning electron microscope (SEM) investigations were carried out using a ThermoFisher Scios 2 Focused Ion Beam (FIB) dual-beam system. The surface roughness and height distribution of the β-Ga_2O_3 and diamond substrate were measured by the atomic force microscope (Bruker Dimension Icon in Germany, AFM).

TEM sample preparation and characterization

The cross-sectional transmission electron microscopy (TEM) samples were fabricated by the ThermoFisher Scios 2 focused ion beam dual-beam system. The samples were polished at low voltages to remove the surface layers at the last steps of the sample preparation. The high angle annular dark field scanning transmission electron microscopy (HAADF-STEM) images, X-ray

spectroscopy (EDX) and electron energy-loss spectroscopy (EELS) mapping were recorded using an aberration-corrected JEOL ARM300F2 operated at 300 kV. TEM images were obtained on a JEOL 2100 TEM at 200 kV. The *in-situ* tensile experiments were carried out with an *in-situ* tensile holder (JFE03, ZepTool) in the FEI Tecnai F20 at 200 kV. The β-Ga_2O_3/diamond sample for tensile test was fabricated by FIB. Both ends of the sample were fixed to the Push-to-Pull (PTP) device by depositing Pt and no slippage occurred during the tensile test. Image processing was performed using DigitalMicrograph (Gatan).

TDTR Method

Time-domain thermoreflectance (TDTR) is a widely recognized pump-probe optical technique for measuring thermophysical properties of various materials [1–3], particularly suitable for determining thermal conductivity (*k*) and thermal boundary conductance (TBC) in nanoscale thin-film structures [4–6]. In a typical TDTR experiment, a mode-locked Ti: sapphire laser (central wavelength 785 nm, repetition rate ~80 MHz) provides a pulsed beam that is divided into pump and probe branches. The pump beam periodically heats the sample surface over a micrometer-scale spot, while the probe beam detects the temperature-induced variation in the reflectance of an aluminum transducer layer at the same position. Details of the optical layout and system calibration are available in previous reports [4,7]. In this work, a 10× objective lens was employed to focus the beams, yielding a spot radius of approximately 6 μm. The average powers of the pump and probe beams were 20 mW and 10 mW, respectively. The β-Ga_2O_3 film was polished by FIB to obtain the TDTR samples.

A multilayer heat diffusion model was employed to simulate the thermal response by incorporating the physical parameters of each layer, including thickness (*t*), *k*, and volumetric heat capacity (*c*). The model produces a theoretical thermal decay profile, which is iteratively fitted to experimental data to extract unknown parameters. In the Al/β-Ga_2O_3/diamond structure, most material properties were either adopted from previous studies or measured independently. The thickness of the Al transducer (~90 nm) was determined using picosecond acoustics [8,9], while its *k* was evaluated from four-point probe measurements combined with the Wiedemann-Franz relation [10]. The *k* of the diamond substrate was calibrated using standard reference samples. The *t* of the β-Ga_2O_3 layer was determined from TEM cross-sectional images. The *c* of the constituent materials was adopted from literature values [11,12]. The remaining unknowns include the TBC of Al/β-Ga_2O_3 interface ($G_{Al/\beta-Ga_2O_3}$), the *k* of the β-Ga_2O_3 layer ($k_{\beta-Ga_2O_3}$), and the TBC of β-Ga_2O_3/diamond interface ($G_{\beta-Ga_2O_3/Diamond}$). These quantities were determined based on the frequency-dependent sensitivity of the TDTR signals to each

parameter. High-frequency modulation (8.7 MHz) enabled reliable extraction of $G_{Al/\beta-Ga_2O_3}$ and $k_{\beta-Ga_2O_3}$, whereas the lower modulation frequency (1.67 and 3.93 MHz) provided enhanced sensitivity to thermal transport at the β-Ga_2O_3/diamond interface, allowing determination of $G_{\beta-Ga_2O_3/Diamond}$.

EELS data acquisition and processing

All the EELS data were acquired on the Nion U-HERMES200 microscope equipped with both a monochromator and aberration correctors, operated at 60 kV with 35-mrad convergence semi-angle and 24.9-mrad collection semi-angle. The electron beam was moved off optical axis with 60 mrad to greatly reduce the contribution of the long-range dipole scattering. The data was collected with 80×5 pixels within the range of 16 nm across the interface. The typical dwell time was 2 s per pixel and the energy dispersion was 0.5 meV/channel. The sample drift was usually within 1.5 nm on this timescale and was corrected afterwards by aligning the interface. The specimen was baked at 160°C for 16 h to further remove the surface contamination layer before EELS experiments.

All EELS spectra were processed by MATLAB code developed by Peng Gao group [13]. Raw EELS spectra are first aligned by their normalized cross section. Then, block-matching and 3D filtering (BM3D) algorithms are applied to remove Gaussian noise [14,15]. The background of EELS was fitted using the modified Pearson-VII function and then subtracted to obtain the phonon signal. The Lucy-Richardson deconvolution algorithm was then employed to ameliorate the broadening effect caused by the finite energy resolution. non-negative matrix factorization (NMF) was performed to decompose the off-axis data into three components, which was already proven effective in processing EELS data [16].

First Principal Calculations

The work of adhesion (W_{ad}) for β-Ga_2O_3 $(\bar{2}01)$, β-Ga_2O_3 (100), β-Ga_2O_3 (001), diamond {111} and the β-Ga_2O_3 $(\bar{2}01)$ /diamond $(\bar{1}11)$ has been investigated based on DFT [17,18]. The exchange-correlation function is selected Perdew-Burke-Ernzerhof (PBE) [19]. The optimized plane-wave cutoff energy is set to 500 eV. The β-Ga_2O_3/diamond interface model includes six-layer β-Ga_2O_3 and five-layer diamond, in which the unsaturated C atoms of diamond slab are passivated by hydrogen atoms. A vacuum of 15 Å was employed on the two side materials to separate the interaction between periodic slabs. The maximum total energy is set to 10^{-5} eV and forces on atoms in the range of -0.03 eV $Å^{-1}$.

The W_{ad} is expressed as the work to hive off a complete interface into two free slabs. The W_{ad} [20–23] is determined by Equation (1):

$$W_{sep} = \frac{(E_\alpha + E_\beta - E_{\alpha/\beta})}{A_i} \quad (1)$$

Where E_α and E_β are the total energy of the isolated α layer and β layer, respectively. $E_{\alpha/\beta}$ devotes total energy of the heterointerface. A_i is the area of the interface. For bulk β-Ga_2O_3 and bulk diamond, the interface is marked as the interior of the crystal.

MD Simulation

The MD simulations were carried out by Graphics Processing Units Molecular Dynamics (GPUMD), using the trained neuro-evolution machine learning potential (NEP) model with training dataset developed by Sun [24]. The simulation system was 53 Å×40 Å×316 Å. Periodic boundary conditions were applied in all directions, and the time step was set as 1 fs. To calculate the phonon density of states (PhDOS) and eigenvector, the system underwent NVE (micro-canonical) ensemble simulation at 300 K for 20 ps. To calculate the spectral distribution of heat current (SHC) and TBC, nonequilibrium molecular dynamics (NEMD) simulations were conducted in four steps. First, to achieve thermal equilibrium, we performed 50 ps simulations in the isothermal-isobaric (NPT) ensemble at 300 K and zero pressure. Then, we switched to NVE ensemble for an additional 50 ps. Next, we carried out NEMD simulations in the NVE ensemble for 150 ps, which is long enough to bring the system to stationary state. Finally, NEMD simulations in the NVE ensemble were carried out for 300 ps to compute the non-equilibrium virial-velocity correlation function, the temperature, and the SHC in direction perpendicular to the interface, for a group of atoms. To minimize thermal coupling between the heat source and sink regions, a fixed boundary condition was imposed along the heat flow direction. To analyze spatial contributions, the simulation domain was partitioned into multiple cells, and the SHC was computed separately for the atoms within each cell.

Figure S1a shows the morphology of β-Ga_2O_3 grown on the diamond (111) surface. It can be seen that the β-Ga_2O_3 film continuously covered the whole diamond surface (3 mm×3 mm). The obtained β-Ga_2O_3 film has a relatively flat surface (Fig. S1b–d), and Ra=51.0 nm (20 μm×20 μm), 9.7 nm (5 μm×5 μm) (Fig. S1e and f).

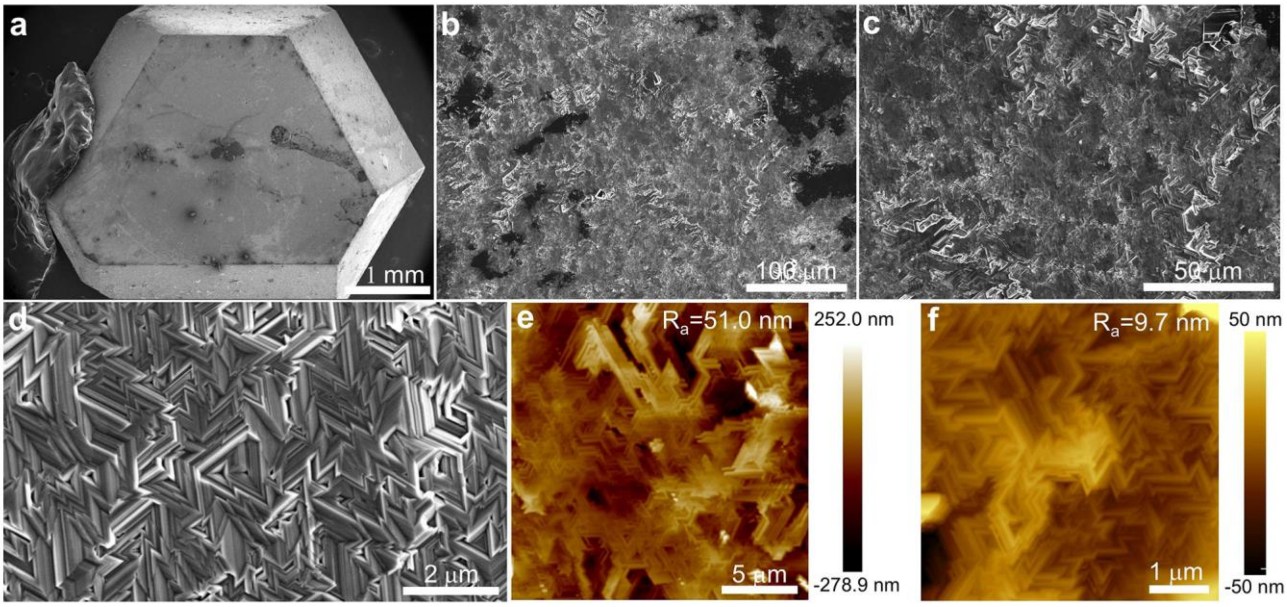

Figure S1. Surface morphology and roughness of the epitaxial β-Ga_2O_3 film on diamond (111). (a–d) SEM images of β-Ga_2O_3 films. (e and f) AFM topography of the β-Ga_2O_3.

Figure S2a shows the enlarged view of the $(\bar{2}01)$ peak from the XRD spectra of Fig. 1e with full width at half maxima (FWHM) =0.08°. Compared with the FWHM concerning the obtained rocking curve (Fig. 1f) of β-Ga_2O_3 on diamond reported in the literature (RF magnetron sputtering (RFMS), CVD, and Metal organic chemical vapor deposition (MOCVD)), it shows a higher crystallization quality (Fig. S2b) [25–29]. The acquired Raman spectrum (Fig. S2c) shows eleven vibrational peaks at 111, 142, 167, 197, 317, 343, 416, 473, 629, 653, and 765 cm^{-1}, which correspond well with the Raman peaks of β-Ga_2O_3 [30].

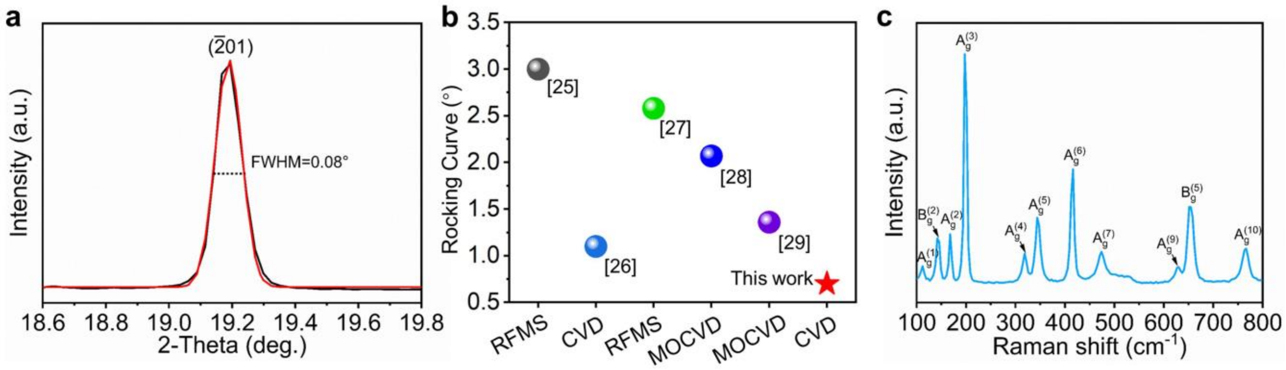

Figure S2. Structural characterization. (a) The enlarged view of the $(\bar{2}01)$ peak from the XRD spectra of Figure 1e. (b) The comparison of the FWHM obtained rocking curve in Figure 1f. (c) Raman characterizations of the epitaxial β-Ga_2O_3 film.

β-Ga_2O_3 film fully covered the entire polished diamond (111) surface, which means Ga has covered the whole diamond surface during the growth process. To evaluate the growth quality of β-Ga_2O_3 on the inner and edge region of the diamond substrate, we prepared two cross-sectional TEM samples at the center (area I) and edge (area II) region of the β-Ga_2O_3 film (Fig. S3a). The atomically-resolved HAADF-STEM characterizations showed that both regions (Fig. S3b and c) present atomic flat interface with directly bonded β-Ga_2O_3.

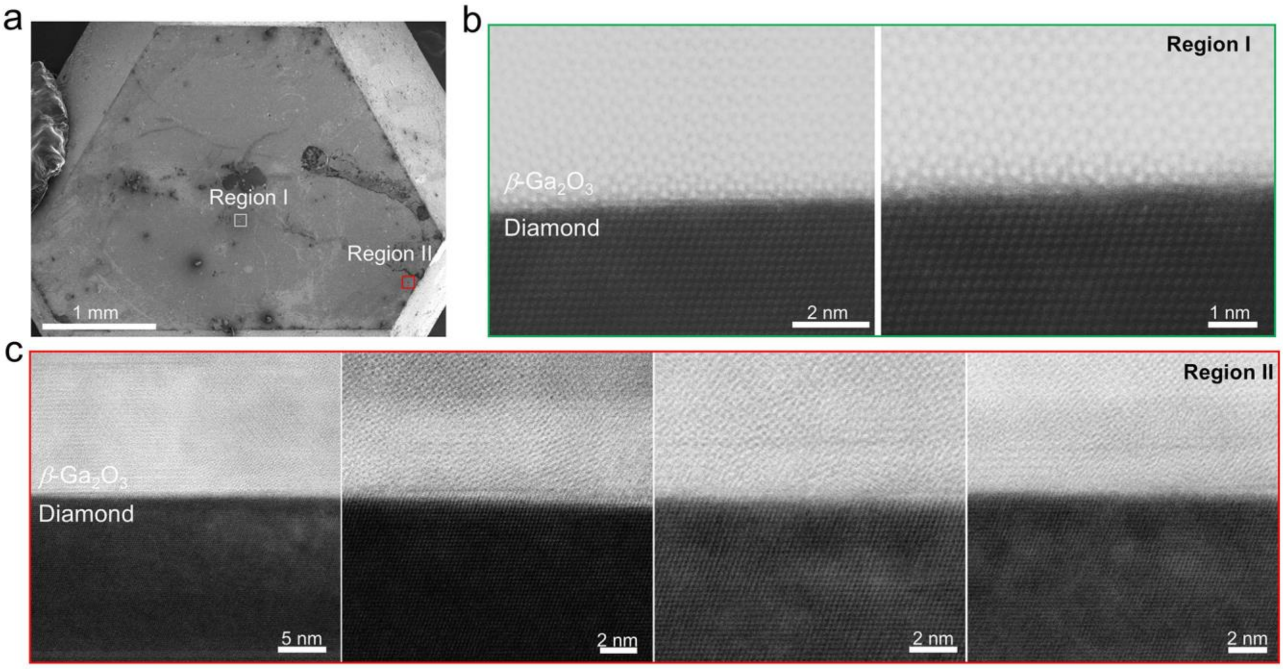

Figure S3. (a) SEM image of the deposited β-Ga_2O_3 on polished diamond (111) surface. Characterizations of the growth quality of β-Ga_2O_3/diamond heterostructure at the (b) center region (region I) and (c) the edge region (region II).

The cross-sectional HAADF-STEM image of the β-Ga_2O_3/diamond interface is shown in Fig. S4a. And the corresponding selected area electron diffraction (SAED) patterns acquired from regions marked by the yellow circles are presented in Fig. S4b–d, respectively. The same feature of the SAED patterns revealed that they possess the same orientation relationships: β-Ga_2O_3 $(\bar{2}01)$ [132] //diamond $(\bar{1}11)$ [110] and β-Ga_2O_3 $(\bar{2}01)$ [010]//diamond $(\bar{1}11)$ [110].

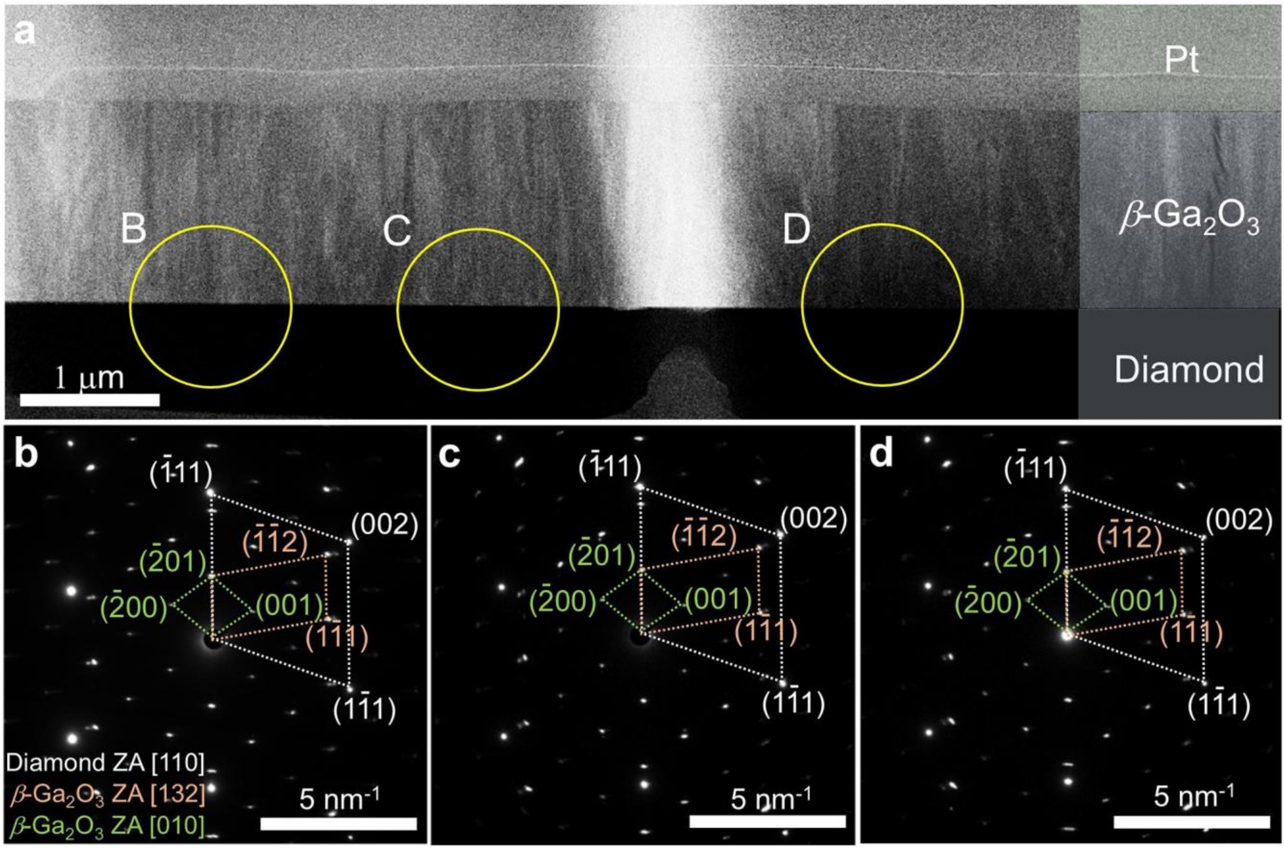

Figure S4. Interfacial structural characterizations. (a) HAADF-STEM image of the β-Ga_2O_3/diamond interface. (b–d) SAED patterns corresponding to the regions marked by the yellow circles.

Figure S5a shows the low-magnification HAADF-STEM image of the β-Ga_2O_3/diamond interface, presenting a sharp interface. Four different interface regions in Fig. S5a were selected to acquire the atomically-resolved HAADF-STEM images, which are shown in Fig. S5b–e, respectively. The two different oriented β-Ga_2O_3 domains can be clearly observed in Fig. S5b. According to Fig. S5b–e, the epitaxial β-Ga_2O_3 film present a sharp interface and no intermediate transition layer was observed. As presented in Fig. S5f–i, by comparing the captured ADF and annular bright-field (ABF) images from the interface regions with the atomic structures of β-Ga_2O_3 and diamond, we construct the interfacial structure and confirmed that O atoms are directly bonded with the C atoms in diamond.

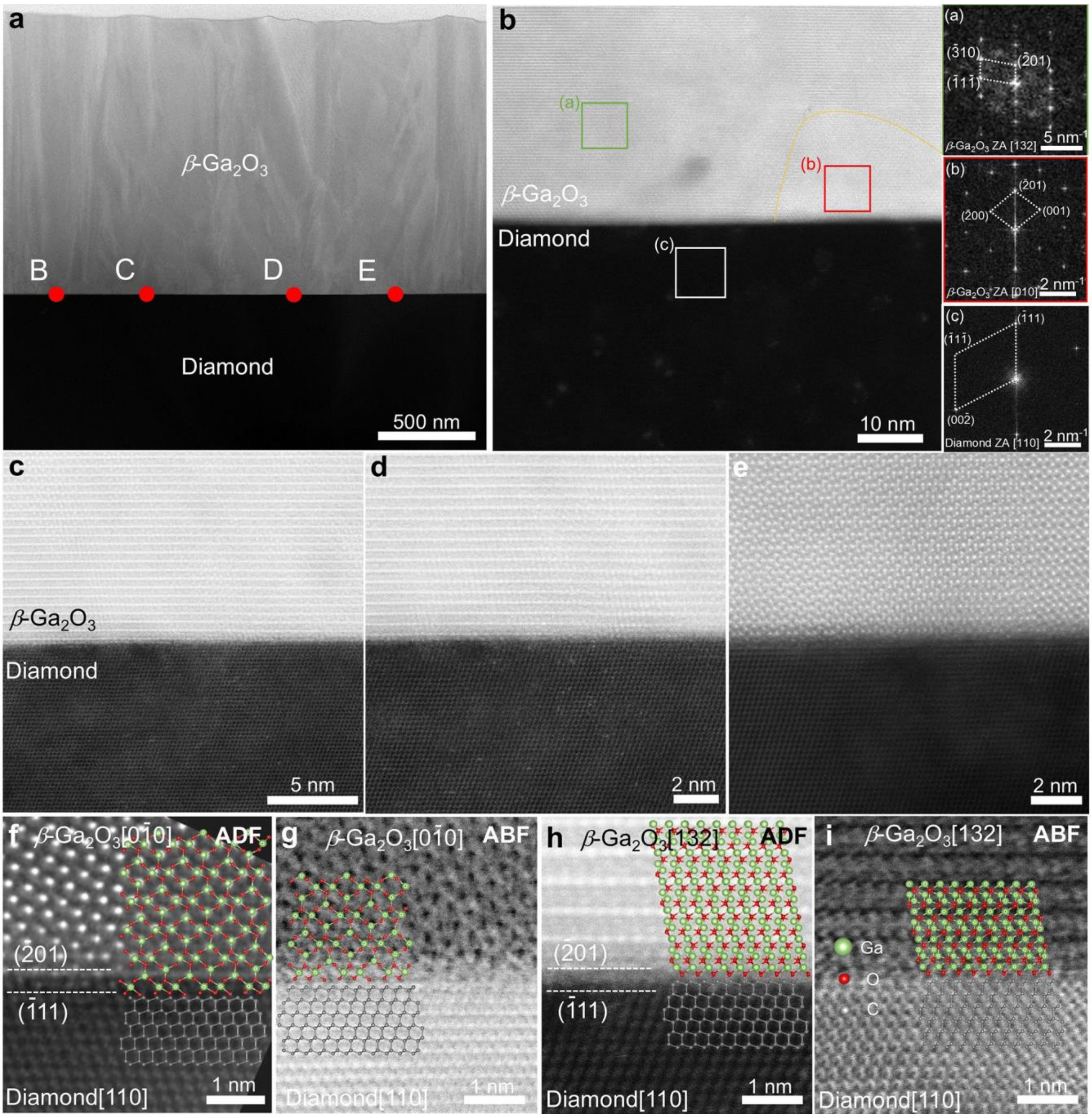

Figure S5. HAADF-STEM images of *β*-Ga_2O_3/diamond interface. (a) Low-magnification HAADF-STEM image of the *β*-Ga_2O_3/diamond interface. (b) Atomically-resolved HAADF-STEM image of the region B marked in a, and the corresponding fast Fourier transform (FFT). (c–e) Atomically-resolved HAADF-STEM images of different regions marked in a. Atomically-resolved (f and g) ADF-STEM and (h and i) ABF-STEM images of the *β*-Ga_2O_3/diamond interface and the corresponding atomic structure model.

Figure S6a presents the atomically-resolved HAADF-STEM image of the β-Ga_2O_3/diamond interface. Fig. S6b presents the acquired EELS mapping from the green shaded region in Fig. S6a. The distribution of C, Ga and O can be clearly observed. Fig. S6c shows the extracted EELS of β-Ga_2O_3 and diamond, with very distinct diamond σ* peak (C-K 291 eV), Ga peak (Ga-L 1142 eV) and oxygen peak (O-K 532 eV).

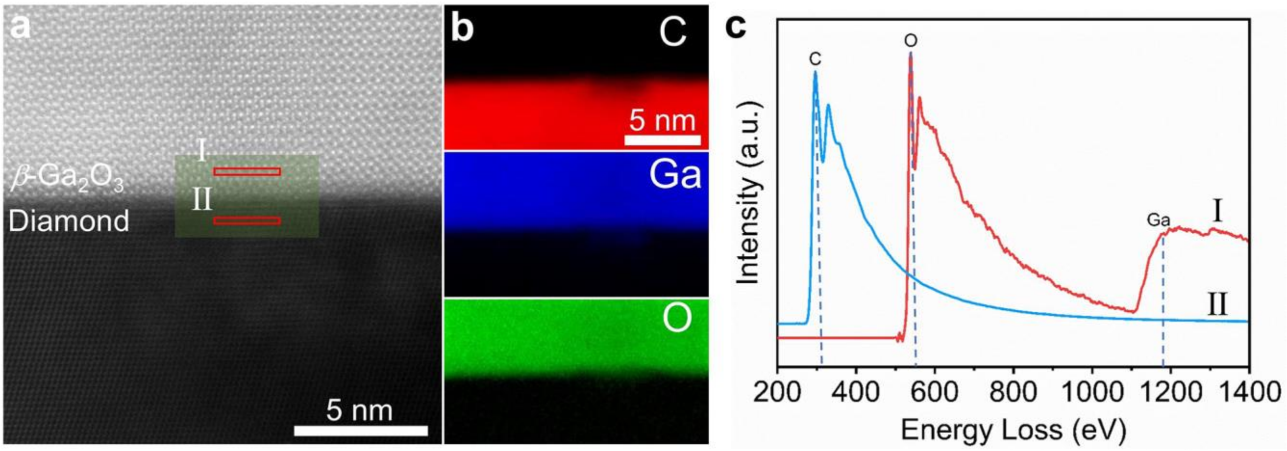

Figure S6. EELS of the β-Ga_2O_3 and diamond. (a) Atomically-resolved HAADF-STEM image of the β-Ga_2O_3/diamond interface. (b) EELS mapping acquired form the green shaded region in a. (c) Extracted EELS spectra from the red framed region (I) (red) and (II) (blue) in a.

By analyzing the morphology of β-Ga_2O_3 film, we found that β-Ga_2O_3 has three orientations, with the included angles being 58.2° and 60.9° respectively (Fig. S7a). As presented in Fig. S7b, by rotating the atomic structure of β-Ga_2O_3 around the direction perpendicular to $(\bar{2}01)$ plane, it can be found that a 58.2° rotation of [010] zone axis (ZA) results in [132] ZA, and another 60.9° rotation gives $[1\bar{3}2]$ ZA.

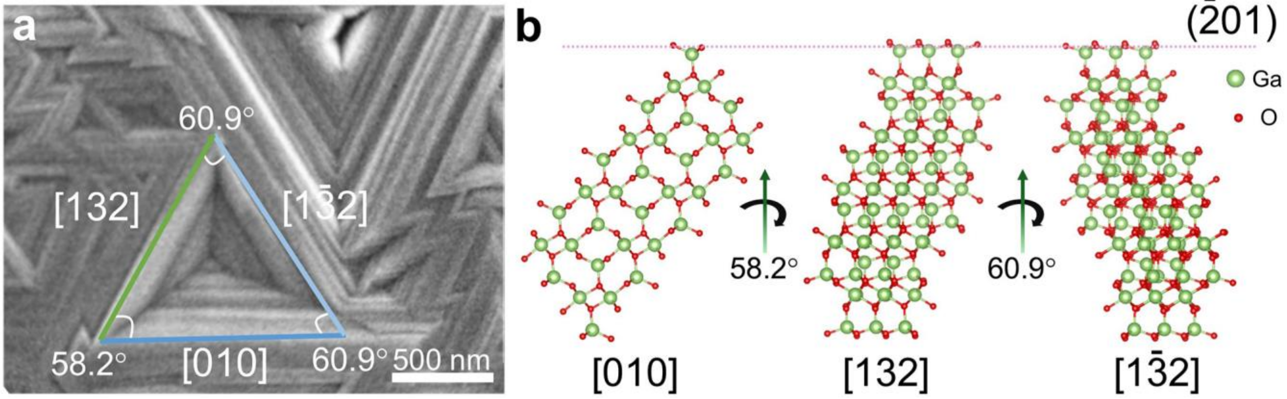

Figure S7. The orientation relationship of β-Ga_2O_3. (a) Surface morphology of the as-grown β-Ga_2O_3 on diamond (111). (b) An atomic structure model of the relationship among [010], [132] and $[1\bar{3}2]$.

Figure S8a and b presents the atomic arrangement of Ga and O in β-Ga_2O_3 $(\bar{2}01)$. Figure S8c presents the atomic arrangement of C in diamond (111). The in-plane atomic arrangement between O on β-Ga_2O_3 $(\bar{2}01)$ in [132]/ [010] direction and C on diamond (111) in [110] direction. On one hand, the C atoms arrangement on the diamond (111) is a symmetric hexagonal shape with a side distance of 4.37 Å, on the other hand, the O atoms arrangement on the β-Ga_2O_3 $(\bar{2}01)$ is an asymmetric hexagonal shape with two different side distances of 4.96 Å and 5.15 Å [25,27,28,31]. Therefore, the lattice mismatch between the O atoms in β-Ga_2O_3 $(\bar{2}01)$ and C atoms in diamond $(\bar{1}11)$ was calculated to be 13.5% ((4.96-4.37)/4.37×100%) ~ 17.76% ((5.15-4.37)/4.37×100%).

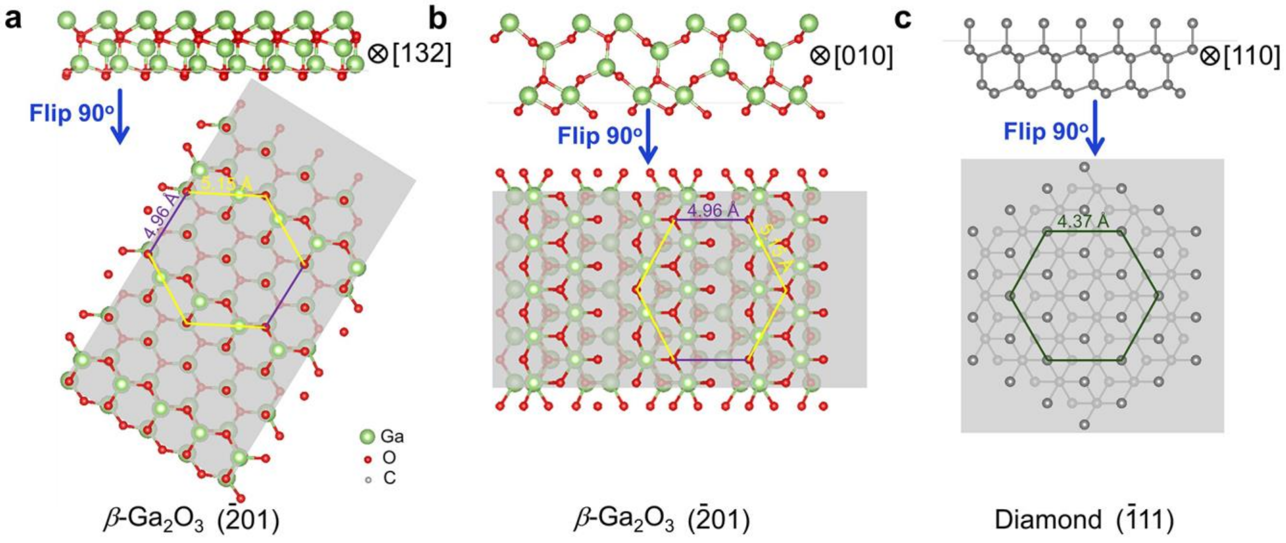

Figure S8. Atomic arrangements of (a and b) β-Ga_2O_3 $(\bar{2}01)$ and (c) diamond $(\bar{1}11)$.

The metal catalyzed diamond etching behavior is generally occurred at temperatures higher than 1000°C. As a contrast, we conducted the same CVD process low-temperature growth of β-Ga_2O_3 on diamond (111) at 600°C, where the metal-diamond reaction cannot happen. According to the cross-sectional TEM characterizations (Fig. S9a), the surface of the diamond (~50 nm thickness) has been crushed into nanocrystals (10 nm in size, Fig. S9b and c), which may be caused by the growth stress. It has been reported that the stress between GaN and diamond can be as high as 20 GPa during the growth process due to the lattice mismatch and thermal mismatch [32]. Moreover, large number of defects were also induced in bulk diamond (Fig. S9d–f) away from the interface region.

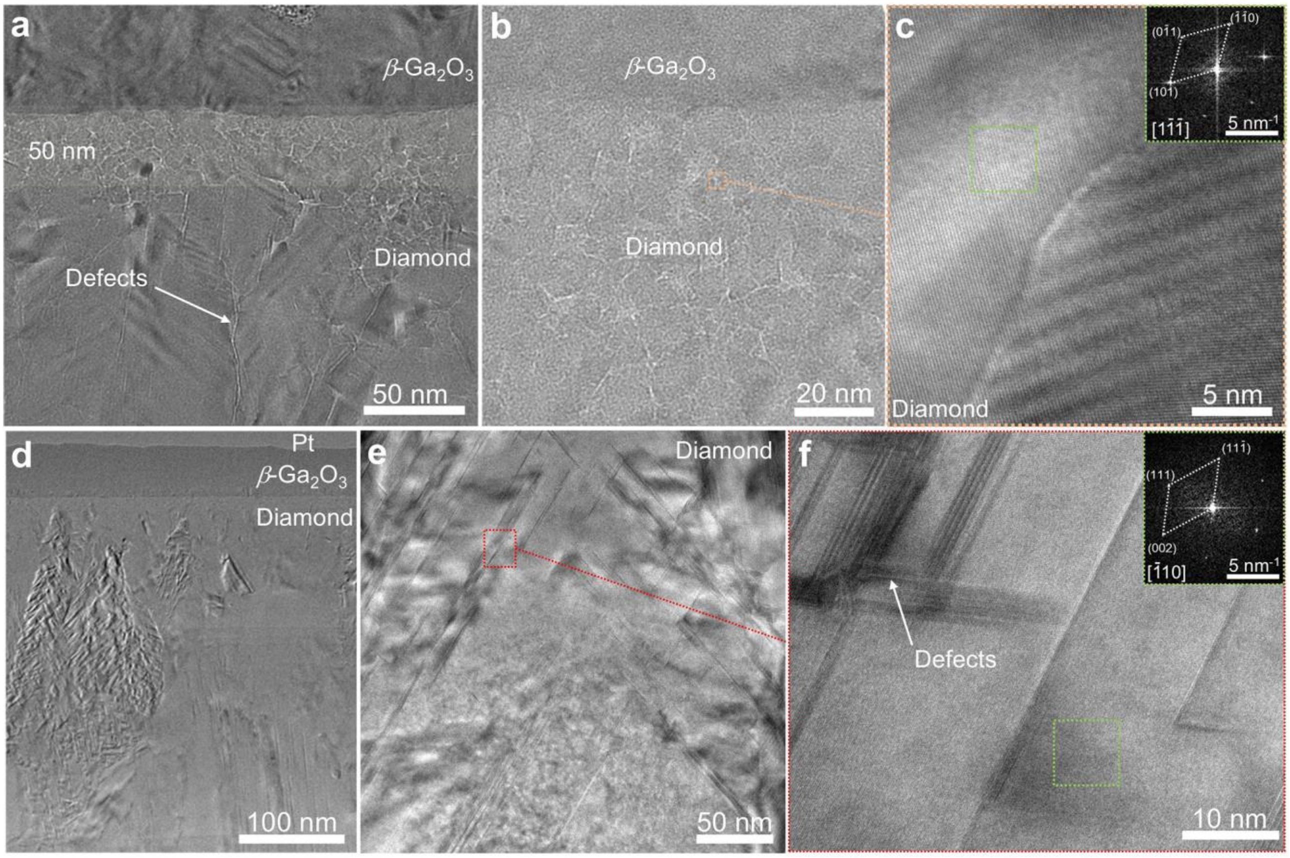

Figure S9. Characterizations of β-Ga_2O_3/diamond interface grown at low-temperature. (a) Low and (b and c) high-mag cross-sectional TEM images of the β-Ga_2O_3/diamond interface. (d–f) TEM images away from the interface.

The surface roughness of diamond (111) substrate can greatly influence the epitaxially growth of *β*-Ga_2O_3. Figure S10a and b show the AFM morphologies of the unpolished and polished single-crystal diamond (111) substrate, with surface roughness of 33.6 nm and 2.87 nm, respectively. For the unpolished substrate, the obtained *β*-Ga_2O_3 grains didn't form a continuous film (Fig. S10c). In contrast, the polished substrate results in a continuous *β*-Ga_2O_3 film (Fig. S10d).

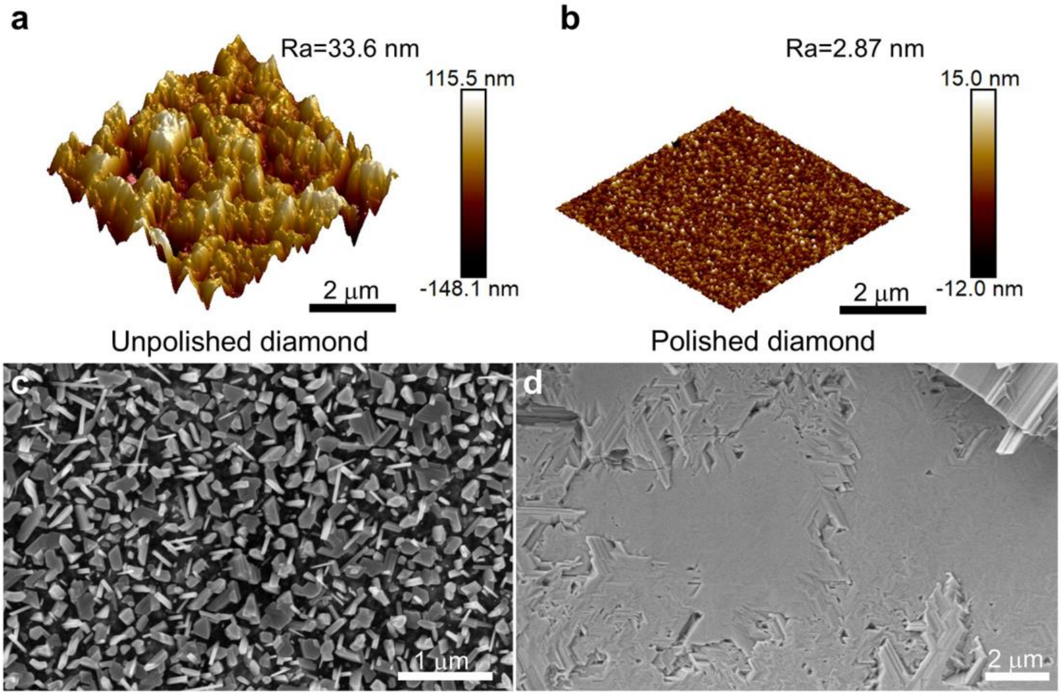

Figure S10. The influence of diamond surface roughness on the growth of the *β*-Ga_2O_3. AFM morphology of the (a) unpolished and (b) polished surfaces of diamond (111) substrate. SEM images of *β*-Ga_2O_3 grown on (c) unpolished and (d) polished single crystal diamond (111) surfaces.

Figure S11a presents the SEM image of the PTP device. The thickness of the FIB fabricated sample was measured to be 167.9 nm (Fig. S11b).

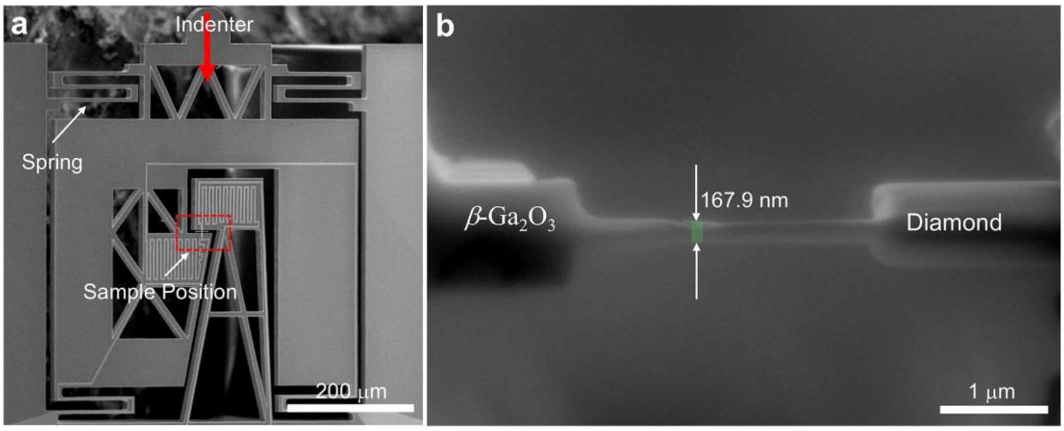

Figure S11. The PTP micro-electro-mechanical system (MEMS) device and the FIB fabricated sample. (a) SEM of the PTP MEMS device used for the *in-situ* TEM tensile test. (b) Side view of the FIB fabricated sample with a thickness of 167.9 nm. The green box indicates the interface area.

Table S1. Comparison of the fracture strength of diamond (and Ga_2O_3) heterostructures.

Heterogeneous Interface	Fracture Strength (MPa)	References
**GaN/diamond**	6.8	[33]
**Si/diamond**	7.78	[34]
**Al(Si)/diamond**	78	[35]
**Cu(Cr)/diamond**	252	[36]
**SiC/diamond**	750	[37]
**β-Ga_2O_3/Si**	6.23	[38]
**β-Ga_2O_3/SiO_2**	7.31	[39]
**Ga_2O_3/SiC**	10	[40]
**β-Ga_2O_3/diamond**	**2090**	**This work**

Figure S12 presents the time-dependent TEM images during the *in-situ* tensile test of the *β*-Ga_2O_3/diamond interface. The fracture occurred near the interface after 4 mins' tension. It should be mentioned that the observed change of the contrast stripes was induced by the applied stress from the PTP device.

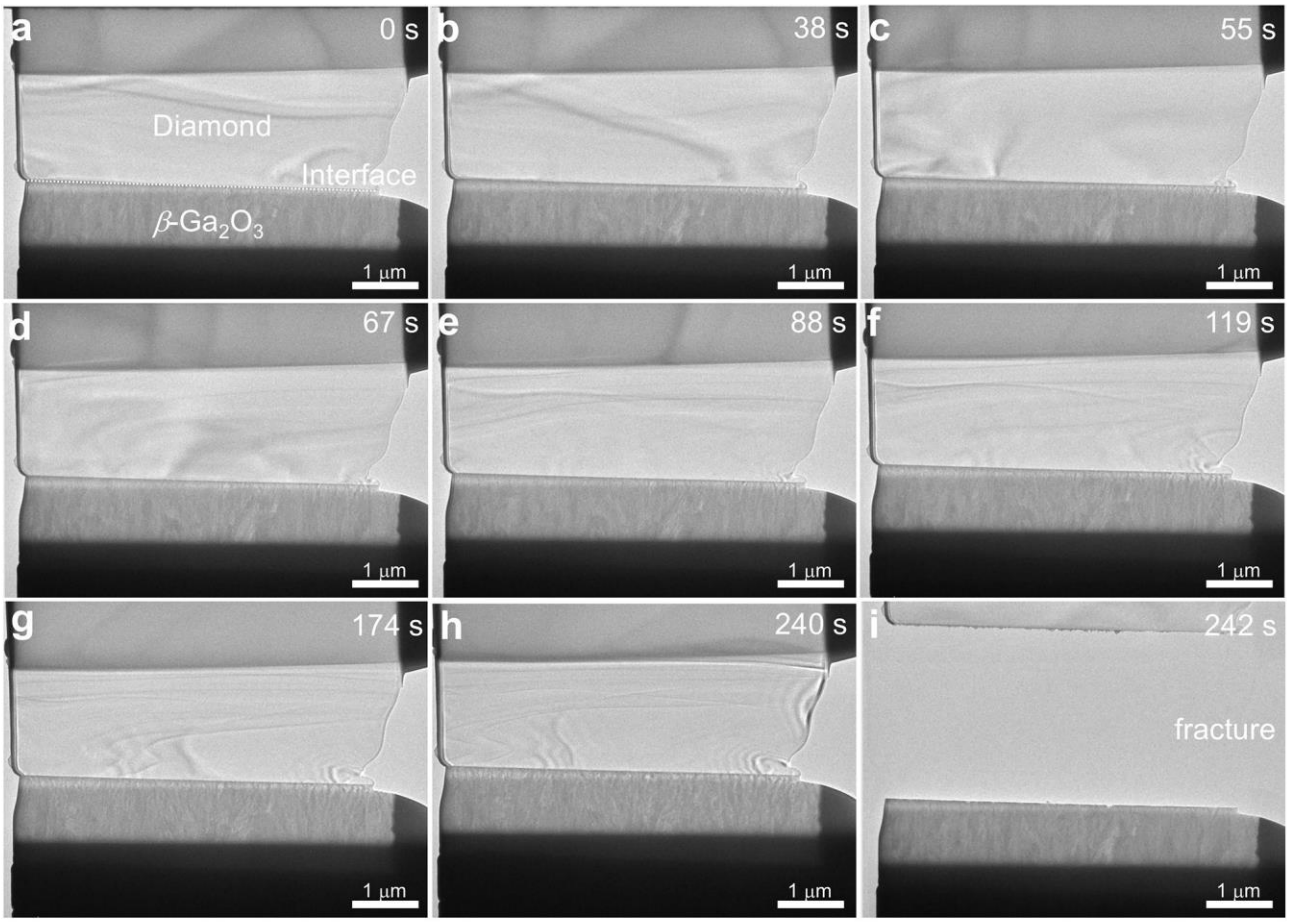

Figure S12. Time-dependent TEM-images in the *in-situ* tensile test.

Figure S13a shows a low magnification TEM image of the fractured surface. Residual β-Ga_2O_3 on the fractured surface can be clearly observed (Fig. S13b–f).

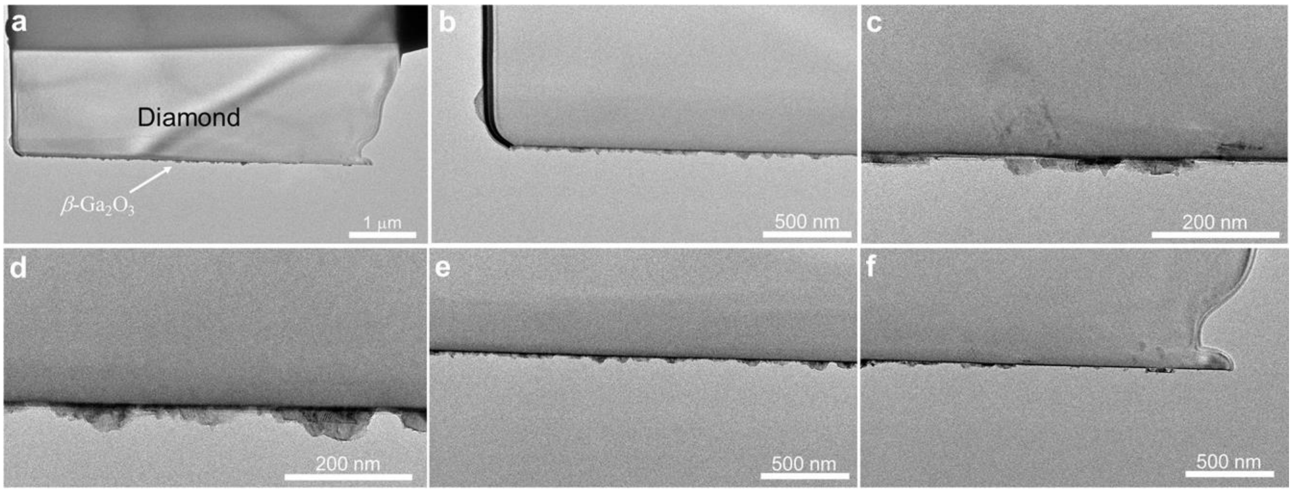

Figure S13. β-Ga_2O_3 remaining on the surface of the diamond after fracture. (a) Low and (b–f) high-mage TEM images of the fractured surface.

To verify whether the zigzag fracture morphology with sharp (200) and (001) cleavage planes is a universal result of the fracture of the $(\bar{2}01)$ oriented β-Ga_2O_3 polycrystalline film, we also carried out similar *in-situ* tensile test on the β-Ga_2O_3/4H-SiC interface with orientation relationship of β-Ga_2O_3 $(\bar{2}01)$ [010]/4H-SiC (0001) [110] (Fig. S14a and b). According to our experimental result, it can be seen that the fracture also occurred at the β-Ga_2O_3 side (Fig. S14c). Our TEM characterizations of the fracture morphology and microstructure (Fig. S14d–f) showed that the fracture in β-Ga_2O_3 present a zigzag morphology with cleavage plane of (001) and (200), which present a similar fracture behavior with our deposited $(\bar{2}01)$ oriented β-Ga_2O_3 on diamond. Therefore, the zigzag with sharp (200) and (001) cleavage planes should be a universal result of $(\bar{2}01)$ oriented polycrystalline β-Ga_2O_3 film.

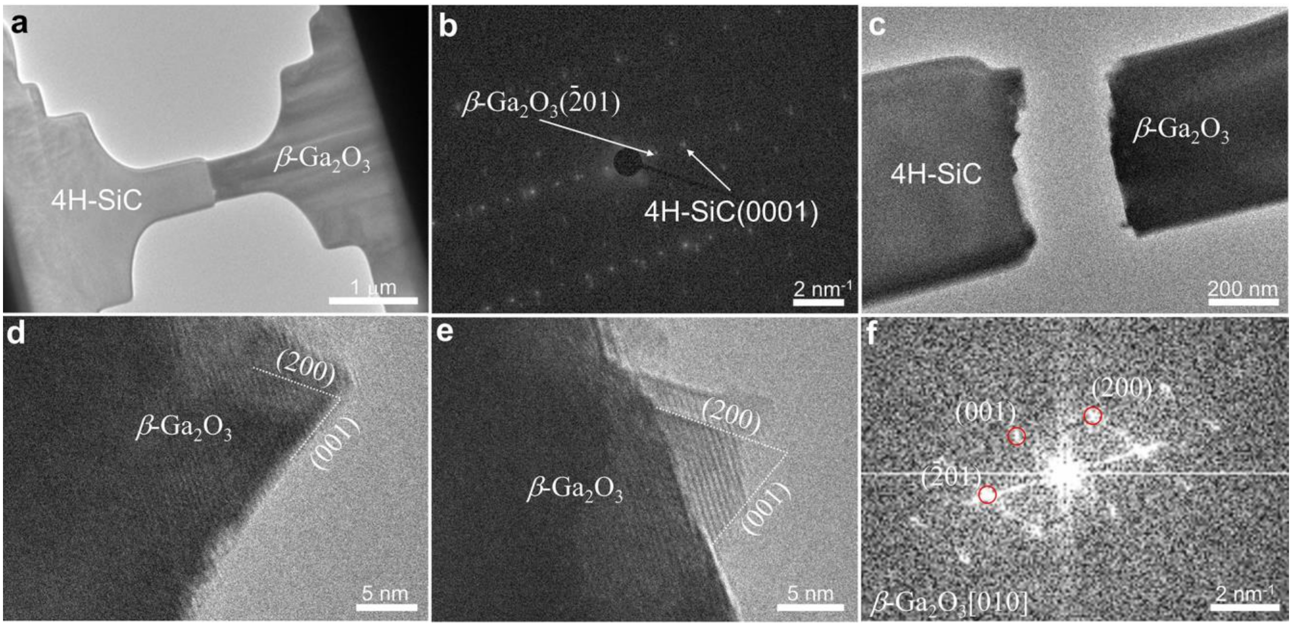

Figure S14 Fracture behavior of the $(\bar{2}01)$ oriented β-Ga_2O_3 film on 4H-SiC. (a) The TEM image and (b) corresponding SAED of the β-Ga_2O_3/4H-SiC sample for *in-situ* mechanical tensile test. (c) Low-mag TEM image of the fractured sample. (d and e) HRTEM images and (f) corresponding FFT of the fractured β-Ga_2O_3.

Figure S15 displays the experimental fitting results for the 213-nm-thick sample, together with the sensitivity analysis of key parameters across two representative thicknesses. The analysis reveals that at 8.7 MHz (Fig. S15a–c), the sensitivity of the *k* of the *β*-Ga_2O_3 layer ($k_{\beta-Ga_2O_3}$) substantially exceeds that of the TBC of the *β*-Ga_2O_3/diamond interface ($G_{\beta-Ga_2O_3/Diamond}$), and the sensitivity of $G_{\beta-Ga_2O_3/Diamond}$ further diminishes with increasing film thickness. Thus, measurements at 8.7 MHz predominantly enable reliable extraction of $k_{\beta-Ga_2O_3}$. In contrast, at 1.67 MHz and 3.93 MHz, the sensitivity to $G_{\beta-Ga_2O_3/Diamond}$ increases, as illustrated for the 213-nm sample in Fig. S15d–f. This allows accurate extraction of $G_{\beta-Ga_2O_3/Diamond}$ at lower modulation frequencies.

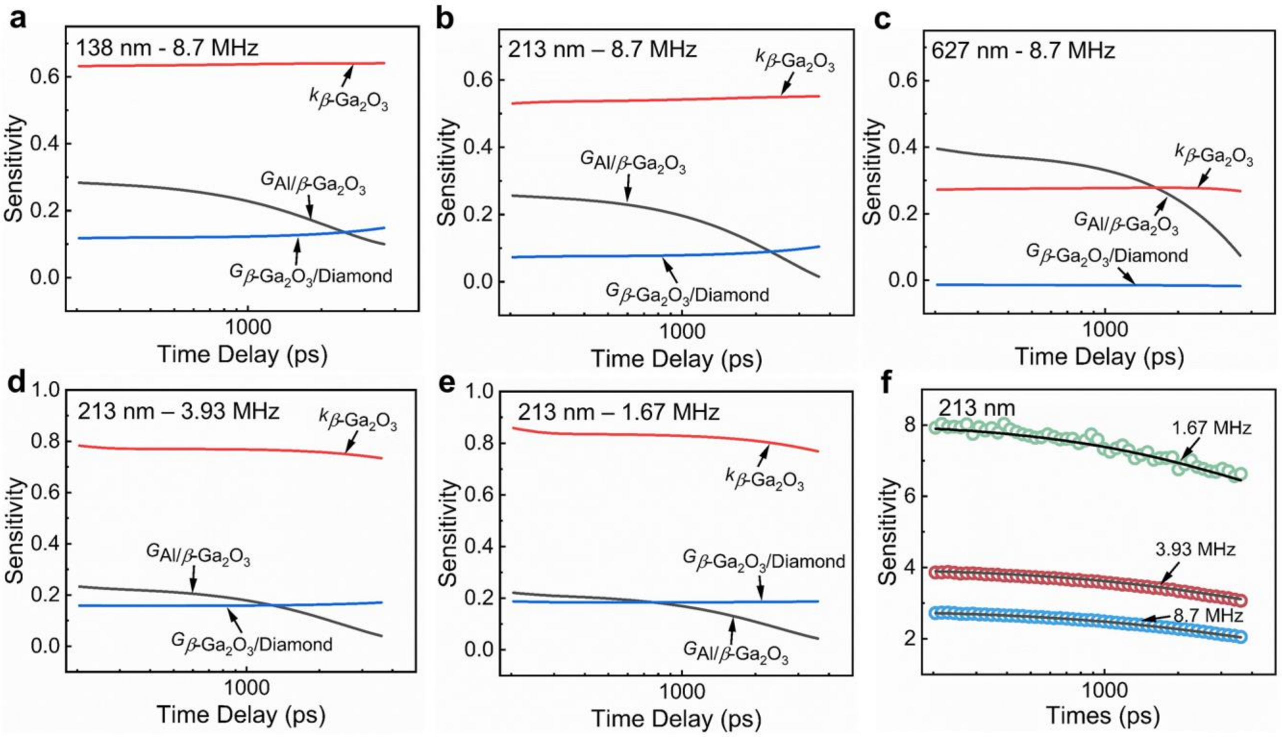

Figure S15. The experimental fitting results for the 213-nm-thick sample, together with the sensitivity analysis of key parameters across two representative thicknesses.

Figure S16 presents the measured line profile of EEL spectra across the β-Ga_2O_3/diamond interface.

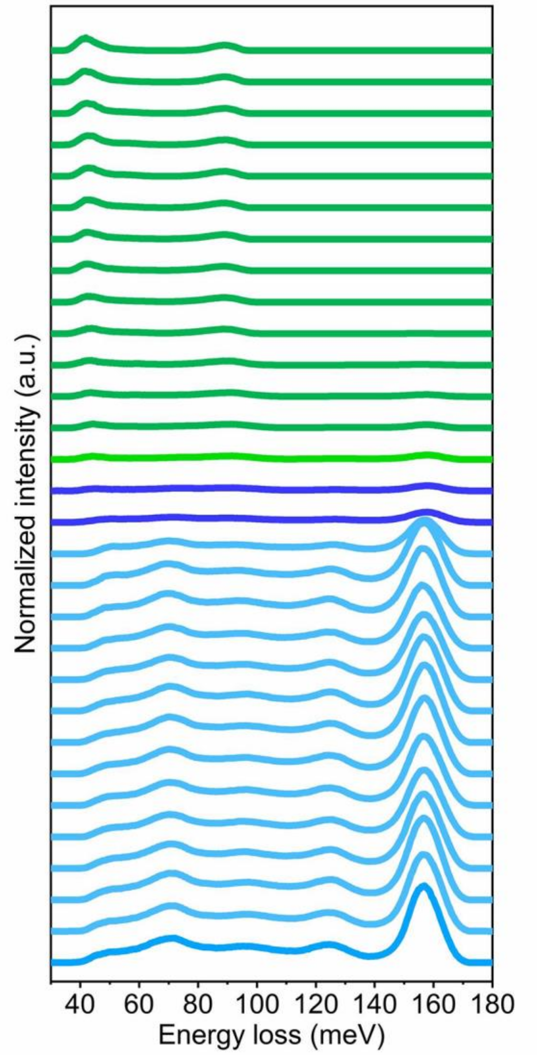

Figure S16. The line profile of EEL spectra across the β-Ga_2O_3/diamond interface with 1/3 nm every stack.

Figure S17 presents the calculated phonon dispersion curves of bulk diamond and β-Ga_2O_3 through GPUMD.

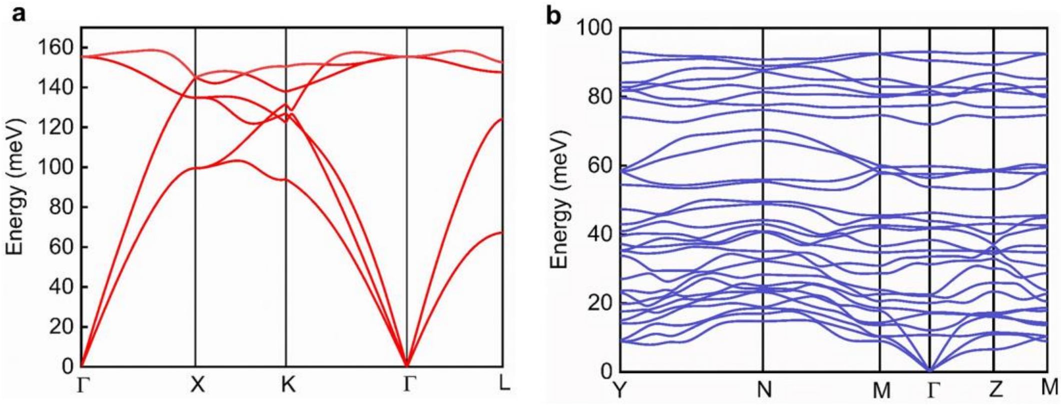

Figure S17. The calculated phonon dispersions for bulk diamond (a) and β-Ga_2O_3 (b).

Figure S18 presents the extracted EEL spectra of components I, II and III by NMF decomposition across the β-$Ga_2O_3\,(\bar{2}01)$/diamond$\,(\bar{1}11)$.

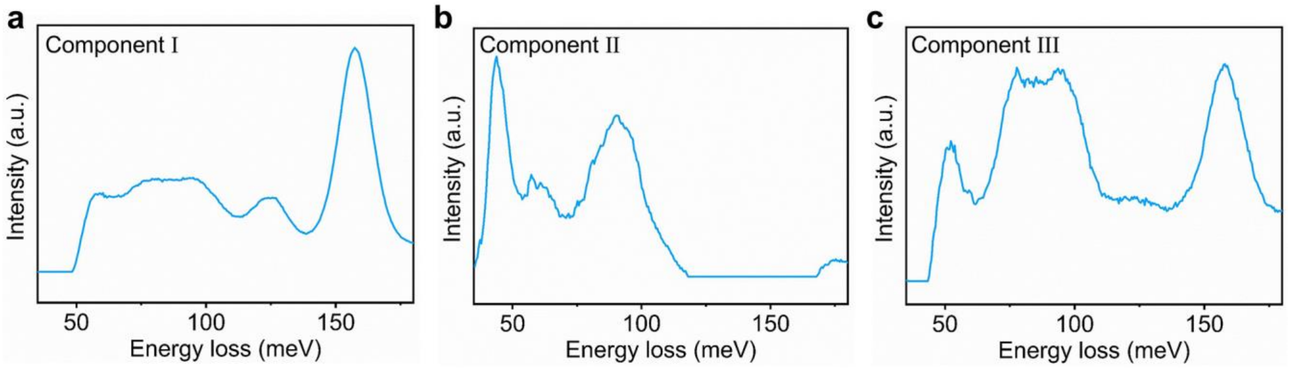

Figure S18. The EEL spectra component of I, II and III across the β-$Ga_2O_3\,(\bar{2}01)$/diamond$\,(\bar{1}11)$ interface.

REFERENCES

1. Kim S, Mujid F, Rai A *et al.* Extremely anisotropic van der Waals thermal conductors. *Nature* 2021; **597**: 660–5.
2. Li M, Wu H, Avery E *et al.* Electrically gated molecular thermal switch. *Science* 2023; **382**: 585–9.
3. Liu C, Si Y, Zhang H *et al.* Low voltage-driven high-performance thermal switching in antiferroelectric $PbZrO_3$ thin films. *Science* 2023; **382**: 1265–9.
4. Zhou J, Yang K, Yang B *et al.* Graphene layer number-dependent heat transport across nickel/graphene/nickel interfaces. *ACS Appl Mater Interfaces* 2022; **14**: 35237–45.
5. Zhou J, Shi C, Zhang Z *et al.* Experimental investigations on thermal transport properties of nanoscale-graphite-film. *J Therm Sci* 2022; **31**: 1008–15.
6. Li Q, Liu F, Hu S *et al.* Inelastic phonon transport across atomically sharp metal/semiconductor interfaces. *Nat Commun* 2022; **13**: 4901.
7. Zhang Z, Chang Z, Fan X *et al.* Pressure dependent thermoreflectance spectroscopy induced by interband transitions in metallic nano-film. *IScience* 2021; **24**: 102990.
8. Thomsen C, Grahn H, Maris H *et al.* Surface generation and detection of phonons by picosecond light pulses. *Phys Rev B* 1986; **34**: 4129–38.
9. Fan X, Zhang Z, Zhu J *et al.* Systematic investigations on doping dependent thermal transport properties of single crystal silicon by time-domain thermoreflectance measurements. *Int J Therm Sci* 2022; **177**: 107558.
10. Ashcroft N and Mermin M. *Solid State Physics, xxi ed.* New York: Holt, Rinehart and Winston, 1976.
11. Guo Z, Verma A, Wu X *et al.* Anisotropic thermal conductivity in single crystal *β*-gallium oxide. *Appl Phys Lett* 2015; **106**: 111909.
12. Lide R. *CRC Handbook of Chemistry and Physics 79th Edition.* Boca Raton: CRC Press, 1998.
13. Shi R, Li Q, Xu X *et al.* Codes for atomic-scale observation of localized phonons at $FeSe/SrTiO_3$ interface. *Nat Commun* 2024; **15**: 3418. Zenodo https://doi. org/10.5281/zenodo.10594261.
14. Dabov K, Foi A, Katkovnik V *et al.* Image denoising by sparse 3-D transform-domain collaborative filtering. *IEEE Trans Image Process* 2007; **16**: 2080–95.
15. Zhou J, Yang Y, Yang Y *et al.* Observing crystal nucleation in four dimensions using atomic electron tomography. *Nature* 2019; **570**: 500–3.
16. Shi R, Li Q, Xu X *et al.* Atomic-scale observation of localized phonons at $FeSe/SrTiO_3$ interface. *Nat Commun* 2024; **15**: 3418.
17. Xu P, Gui X, Zhang X *et al.* Wetting and interfacial behavior of Al-Ti/4H-SiC system: A combined study of experiment and DFT simulation. *Ceram Int* 2021; **47**: 32545–53.
18. Wang L, Dhar S, Feldman L *et al.* Nitrogen-induced changes in the electronic and structural properties of 4H-SiC (0001)/SiO_2 interfaces. *Phys Status Solidi* 2021; **259**: 2100224.
19. Liu B and Yang J. Mg on adhesion of Al (111)/3C-SiC (111) interfaces from first principles study. *J Alloys Compd* 2019; **791**: 530–9.
20. Wang Y, Li M, Peng P *et al.* Preferred orientation at the Al/graphene interface: First-principles calculations and experimental observation. *J Alloys Compd* 2022; **900**: 163304.

21. Wang Y, Wang W, Fang S *et al.* The interface characteristics of TiN (100)/MgO (100) multilayer on oxidized Si (100) substrate via first-principle calculations and experimental investigation. *Mol Simul* 2020; **47**: 552–9.
22. Liu X, Dong H, Lv X *et al.* The adhesion, stability, and electronic structure of γ-TiAl/VN interface: a first-principle study. *Appl Phy A-Mater* 2018; **124**: 531.
23. Sun W, Zhang L, Huang X et al. Works of separation for ZnO(0001)/ ZrO_2(111) interfaces: A first-principle study. *Comput Mater Sci* 2017; **136**: 157–62.
24. Sun Z, Zhang D, Qi Z *et al.* Insight into interfacial heat transfer of β-Ga_2O_3/diamond heterostructures via the machine learning potential. *ACS Appl Mater Interfaces* 2024; **16**: 31666–76.
25. Kusaba T, Sittimart P, Katamune Y *et al.* Heteroepitaxial growth of β-Ga_2O_3 thin films on single crystalline diamond (111) substrates by radio frequency magnetron sputtering. *Appl Phys Express* 2023; **16**: 105503.
26. Karim M, Chen Z, Feng Z *et al.* Two-step growth of β-Ga_2O_3 films on (100) diamond via low pressure chemical vapor deposition. *J Vac Sci Technol A* 2021; **39**: 023411.
27. Misono I, Nekita S, Gao H *et al.* Heteroepitaxial growth of β-Ga_2O_3 on diamond (111) via radio frequency magnetron sputtering: mechanistic insights from scanning/transmission electron microscopy. *Small* 2025; **21**: e07322.
28. Nandi A, Cherns D, Sanyal I *et al.* Epitaxial growth of (-201) β-Ga_2O_3 on (001) diamond substrates. *Cryst Growth Des* 2023; **23**: 8290–5.
29. Nandi A, Mandia R, Sanyal I *et al.* Adherent β-Ga_2O_3 thin films on single crystal diamond (001) substrates enabled by $(Al_xGa_{1-x})_2O_3$ buffer layers. *APL Mater* 2025; **13**: 071108.
30. Das D, Gutierrez G, Ramana C. Raman spectroscopic characterization of chemical bonding and phase segregation in Tin (Sn)-incorporated Ga_2O_3. *ACS Omega* 2023; **8**: 11709–16.
31. Seo D, Kim S, Kim H *et al.* Heteroepitaxial growth of single-crystalline β-Ga_2O_3 on GaN/Al_2O_3 using MOCVD. *Cryst Growth Des* 2023; 23: 7090–4.
32. Yu X, Li Y, He R *et al.* Mechanical regulation to interfacial thermal transport in GaN/diamond heterostructures for thermal switch. *Nanoscale Horiz* 2024; **9**: 1557–67.
33. Wang K, Ruan K, Hu W *et al.* Room temperature bonding of GaN on diamond wafers by using Mo/Au nano-layer for high-power semiconductor devices. *Scr Mater* 2020; **174**: 87–90.
34. Wang F, Wang K, Chen G *et al.* Room temperature bonding of diamond/Si with Mo/Au interlayers in atmospheric air. *Diam Relat Mater* 2023; **135**: 109844.
35. Wu J, Zhang H, Zhang Y *et al.* Enhanced mechanical properties in Al/diamond composites by Si addition. *Rare Met* 2016; **35**: 701–4.
36. Xie Z, Guo H, Zhang X *et al.* Tailoring the thermal and mechanical properties of diamond/Cu composites by interface regulation of Cr alloying. *Diam Relat Mater* 2021; **114**: 108309.
37. Zhang Y, Hsu C, Zhao Y *et al.* *In situ* measurement of diamond/SiC interfacial strength. *Microsc Microanal* 2019; **25**: 848–9.
38. Ma X, Mu W, Hou T *et al.* Low-cost O_2 plasma activation assisted direct bonding of β-Ga_2O_3 and Si substrates in air. *Mat Sci Semicon Proc* 2024; **179**: 108512.
39. Ma X, Mu W, Hou T *et al.* Low-temperature bonding process of β-Ga_2O_3/SiO_2 activated by oxygen plasma

(Chinese). *J Chin Ceram Soc* 2024; **52**: 3273–82.

40. Lin C, Hatta N, Konishi K *et al.* Single-crystal-Ga_2O_3/polycrystalline-SiC bonded substrate with low thermal and electrical resistances at the heterointerface. *Appl Phys Lett* 2019; **114**: 032103.
